# Artificial Intelligence-Driven Natural Product Discovery for Cancer Metastasis and Chemoresistance: From Computational Prediction to Preclinical Validation

**DOI:** 10.3390/cancers18050719

**Published:** 2026-02-24

**Authors:** Mohamed Ali Hussein, Gnanasekar Munirathinam

**Affiliations:** 1 Institute of Global Health and Human Ecology, School of Sciences and Engineering, The American University in Cairo, New Cairo 11835, Egypt; mohamed_hussein@aucegypt.edu; 2 Department of Biomedical Sciences, College of Medicine, University of Illinois, Rockford, IL 61107, USA

**Keywords:** natural products, artificial intelligence, chemoinformatics, AI-facilitated natural products discovery, metastasis, chemoresistance, drug discovery, graph neural networks, network pharmacology

## Abstract

Cancer metastasis and chemoresistance are primary reasons for cancer-related mortality. Current therapeutic options rely mostly on single-target drugs, which often fail to exhibit long-lasting remission of the disease progression due to the complexity of metastasis and resistance mechanisms. Natural products (NPs) possess inherent structural diversity, rendering them suitable as multi-target agents. The utilization of NPs is often impeded in treating complex diseases such as cancer, even though approximately 65% of approved anticancer drugs are NP derivatives, or synthetic derivatives containing NP-pharmacophores, due to various factors, including poor aqueous solubility and variable oral bioavailability, structural complexity, synthetic inaccessibility, and stereochemical diversity that confounds structure–activity relationship analyses. This review discusses how integrating artificial intelligence (AI) and machine learning (ML) with chemoinformatics can identify, prioritize, and experimentally validate NPs, potentially paving the way for new drugs that address intricate processes such as metastasis and resistance. We summarize the recent computational advances in the field, including graph neural networks, attention mechanisms, Siamese networks, virtual screening, and network pharmacology. These advancements address ADMET optimization, molecular representation, virtual screening, network pharmacology, and experimental validation. We emphasize how each of these approaches tackles the unique challenges associated with NPs. We contextualize our review within the specific challenges presented by the chemical space of NPs. Additionally, we analyze real-world case studies of successful AI-assisted NP discovery and categorize the quality of evidence into three levels: Level A, which includes in vivo efficacy with mechanistic details; Level B, which consists of in vitro validation of mechanisms and phenotypes; and Level C, which represents computational hypotheses that are awaiting experimental verification. Additionally, we propose an operational framework for selecting suitable AI methodologies based on available data, target characterization, and validation resources. Finally, we emphasize the limitations and future directions in AI-facilitated NP discovery.

## Simple Summary

Cancer is a major public health burden, with substantial impacts on survival and quality of life. It becomes particularly life-threatening when it spreads to other organs or develops resistance to treatment. Single-target therapies remain common in current cancer care, yet this strategy often fails to deliver long-term disease control. Natural compounds show promise as cancer therapies because they can simultaneously affect multiple biological targets. However, their complex structures, poor solubility, and less-than-ideal drug-like properties have slowed progress toward clinical approval. This review discusses how artificial intelligence and computational tools can help overcome these challenges by identifying, optimizing, and prioritizing natural compounds with anticancer potential. In this article, we have reviewed the recent computational methods, showcase real-world examples of successful discoveries, and offer a practical guide for choosing the best approach with the available data and resources. These advances could accelerate the development of more effective treatments for patients with aggressive or treatment-resistant cancers.

## 1. Introduction

Cancer is a significant global health issue with over two million new cases and around 618,000 deaths in the United States in 2025 [1]. Despite advances in treatment options, metastasis and drug resistance remain the leading causes of cancer-related mortality [2,3]. Metastasis is a complex, multi-step process in which tumor cells acquire invasive capabilities through epithelial-to-mesenchymal transition (EMT), facilitating the invasion of primary tumor cells into the basement membrane, dissemination to the bloodstream, extravasation to distant sites to form colonies, and adaptation to the new tissue environment to form distant metastases [3]. Likewise, chemoresistance has a similar mechanistic complexity involving the synchronization of various cellular processes and pathways that interact promptly to sustain the resistance phenotype. This necessitates coordination between several key players, including the increased expression of ATP-binding cassette (ABC) efflux transporters, the expansion of cancer stem cells (CSCs), metabolic changes, modifications in the tumor microenvironment, immune suppression, and the activation of alternative survival pathways [4,5,6].

The current therapeutic options primarily focus on targeted agents that address specific oncogenic drivers or upregulated pathways. Although these agents initially elicit significant clinical remission, they often fall short due to various reasons, which necessitate the use of intensive chemotherapeutic agents associated with deleterious side effects [7]. On the other hand, natural products (NPs) offer a potential avenue to tackle this problem by utilizing their inherent polypharmacological properties, which target multiple pathways simultaneously with potentially more favorable profiles [8,9,10]. This positions NPs as a compelling alternative. Indeed, Newman and Cragg’s comprehensive analysis of approved oncology drugs from 1981–2019 found that approximately 65% of small-molecule anticancer agents were NPs, their derivatives, or synthetic compounds incorporating NP-derived pharmacophores. Specifically, 33.5% were classified as NPs or direct derivatives (N), with an additional 31.4% representing synthetic compounds with NP pharmacophores or NP mimics (S*/NM) [11]. For instance, among the most commonly used anticancer agents in the clinical setting today are paclitaxel, irinotecan, topotecan, vincristine, vinblastine, and etoposide, all of which are derived from NPs [12,13]. Beyond these approved drugs, thousands of NPs show promising effects against cancer in preclinical activity studies, with many reported to influence invasion, metastasis, and resistance mechanisms [11,14,15]. This review focuses on NPs derived from plants, marine organisms, or microbes, including both primary and secondary metabolites. It highlights bioactive compounds traditionally used in medicine or identified through screening for biological activity. The review covers various resistance mechanisms such as: (1) drug efflux mediated by ABC transporters like ABCB1/P-glycoprotein, ABCC1/MRP1, and ABCG2/BCRP; (2) EMT and related transcription factors (SNAI1/Snail, TWIST, ZEB1/2); (3) CSC markers, including CD44, CD133, and ALDH; (4) alterations in DNA repair pathways, especially homologous recombination and nucleotide excision repair; (5) metabolic reprogramming involving glycolysis, glutaminolysis, and oxidative phosphorylation; and (6) resistance mediated by the tumor microenvironment, such as hypoxia, immune suppression, and stromal remodeling.

The complex structures of NPs—which are often seen as disadvantages—may actually be beneficial for tackling complex diseases such as cancer, where a multi-target effect is needed to overcome emerging resistance to conventional chemotherapy. NPs can simultaneously target multiple biological pathways, a phenomenon known as polypharmacology [16]. This allows concurrent targeting of numerous targets involved in metastasis and/or drug resistance—for instance, inhibiting EMT transcription factors while reducing efflux transporter expression, or targeting CSC self-renewal in conjunction with eliciting a cytotoxic effect. Several NPs demonstrate this multi-target capability, as depicted by agents such as kaempferol, which prevents TGF-β1-induced SNAI1/Snail expression by blocking Smad3 from binding to the Snail promoter [17]. Withaferin A has demonstrated modulatory effects on multiple resistance-associated pathways, including TGF-β/Smad2/3, PI3K/Akt, and NF-κB, across preclinical cancer models, with impacts on EMT markers and drug efflux mechanisms. However, the specific contexts and magnitudes of these effects vary across tumor types and experimental systems [18,19]. This exemplifies how NPs can address resistance by coordinating effects across interconnected cellular processes. Despite their therapeutic potential, NPs remain underexplored in modern drug discovery applications due to several factors, including: (i) poor aqueous solubility and variable oral bioavailability; (ii) structural complexity that challenges traditional molecular modeling approaches; (iii) synthetic inaccessibility complicating lead optimization; and (iv) stereochemical diversity that confounds structure–activity relationship analyses [20,21,22]. Consequently, this shifted the focus toward synthetic small-molecule drug discovery, largely overlooking the chemical space of NPs. However, this trend is rapidly changing due to the recent advances in artificial intelligence (AI) and machine learning (ML), combined with the expansion of NP databases and improved analytical methods for structural characterization, all of which are catalyzing systematic exploration of promising NPs [23,24]. AI/ML approaches, in particular, could address challenges related to NP, stereochemical complexity, scaffold novelty, and data scarcity, thereby facilitating their use and exploration [14,25,26].

Despite evident benefits, a paradox remains: if NPs are genuinely superior multi-target agents, why has their direct application in approved anticancer drugs declined recently? Newman and Cragg’s review reveals that the share of compounds labeled as natural products (N) or natural-product derivatives (ND) fell from 53.3% (40 of 75; 1946–1980) to 33.5% (62 of 185; 1981–2019), despite the ongoing influence of NPs on NP-inspired synthetic classes [11]. This trend underscores development challenges that structural diversity alone cannot address. First, off-target effects in normal tissues can narrow therapeutic windows; for example, microtubule-targeting agents such as paclitaxel and vincristine are strongly linked to peripheral neuropathy, likely due to disruption of neuronal microtubule processes, including axonal transport [27,28]. Second, the industry’s shift toward target-based discovery and high-throughput screening—centered on synthetic and combinatorial libraries—has systematically deprioritized traditional NP workflows, which are slower and more variable during extraction and fractionation [29,30]. Third, structure–activity relationship (SAR) and Chemistry, Manufacturing, and Controls (CMC) optimization are often simpler for modular synthetic scaffolds than for stereochemically complex NPs, which may require multi-step synthesis or constrained semisynthesis routes [30]. Fourth, although polypharmacology can be advantageous, demonstrating that a multi-target profile is selective—covering multiple disease-relevant targets rather than being promiscuous—is crucial to limit toxicity, especially in oncology, where some mechanisms of efficacy and toxicity involve shared target classes. For instance, anthracyclines—topoisomerase II inhibitors—are known to be cardiotoxic, with strong evidence indicating that TOP2β-dependent DNA damage in heart cells contributes to cardiotoxicity [31]. Consistent with this, Antolín et al. [32] demonstrated that drugs often engage multiple targets beyond their primary one. Rationally exploiting polypharmacology—rather than viewing it solely as a risk—may be vital for addressing tumor heterogeneity and adaptive resistance in precision oncology [32]. The main challenge remains achieving selective polypharmacology: developing multi-target profiles that are therapeutically beneficial while avoiding toxicity. AI can assist by predicting selectivity, prioritizing target combinations with favorable efficacy–toxicity balances, and guiding semisynthesis and analog development; however, comprehensive prospective validation—particularly for toxicity prediction and network-level selectivity—is still limited. Therefore, these capabilities should be regarded as emerging rather than fully resolved.

**Figure 1:**
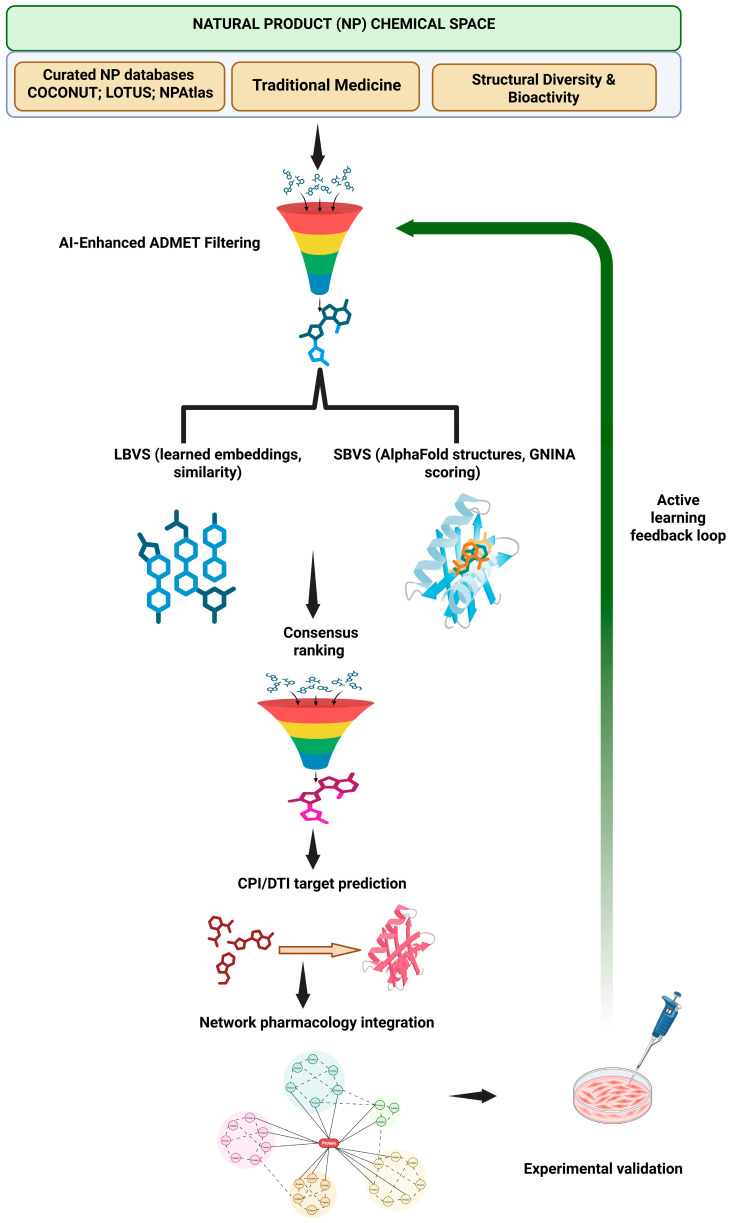
An integrated AI-driven approach to NPs related to metastasis and chemoresistance. A diverse chemical space is compiled from repositories such as COCONUT and LOTUS, along with traditional medicine sources, and standardized, with duplicates removed, before screening. NPs that pass initial screening undergo AI-based developability assessment (including ADMET and safety scoring) to identify compounds with favorable properties. Simultaneously, ligand-based virtual screening (using learned molecular embeddings and similarity searches) and structure-based approaches (docking and ML rescoring on targets derived from experimental or AlphaFold models) provide evidence of activity. These results are integrated through consensus ranking to select top candidates. The predicted hits are further analyzed for secondary targets and off-target effects using compound–protein and drug–target interaction models, and network pharmacology is combined to link targets to pathways involved in metastasis and resistance, thereby aiding target deconvolution. The most promising candidates are then tested in relevant metastatic and resistant models. Outcomes from these experiments inform iterative model updates—adjusting priors, scores, and compound choices—forming a feedback loop. Throughout, key decision points and ML/AI methods are emphasized at each stage. Created with BioRender.com.

This review examines how AI-driven strategies are overcoming traditional obstacles in NP drug discovery, with a focus on applications related to metastasis and chemoresistance. We focus on computational techniques spanning ADMET optimization, molecular representation, virtual screening, and network pharmacology, as well as experimental validation, and emphasize how each addresses challenges unique to NPs. A key goal is to establish a decision framework for choosing methods based on the available data, target characterization, and validation resources. Case studies illustrate successful AI-facilitated discovery of NPs targeting metastatic and resistant phenotypes, followed by the experimental validation frameworks required for translating these findings into practical applications (Figure 1).

### From Computational Prediction to Experimental Validation: A Translational Framework

The application of AI in NP discovery for metastasis and chemoresistance follows a structured translational pathway, which this review systematically organizes to elucidate. The workflow generally advances through five interconnected stages: (1) Computational prioritization, AI-enabled virtual screening (Section 4), network pharmacology (Section 5), and ADMET prediction (Section 2) identify promising candidates from extensive NP libraries; (2) Target validation, involving biophysical confirmation of compound–protein interactions (CPI) through techniques such as surface plasmon resonance (SPR), isothermal titration calorimetry (ITC), or cellular thermal shift assays (CETSA) (Section 7); (3) Phenotypic validation, assessing effects on resistance-relevant phenotypes including EMT markers, CSC populations, drug efflux capacity, and invasion/migration using two-dimensional and three-dimensional cell culture models; (4) Mechanistic confirmation, elucidating pathway-level effects through transcriptomics, proteomics, and phosphoproteomics to verify AI-predicted mechanisms of action; and (5) In vivo efficacy assessment, demonstrating anti-metastatic or chemosensitizing activity in orthotopic, patient-derived xenograft (PDX), or genetically engineered mouse models (GEMMs), ideally with pharmacokinetic profiling.

Each stage incorporates feedback loops—a concept central to active learning (Section 7)—wherein experimental results refine computational models, thereby enhancing subsequent predictions. For instance, validated hits inform model retraining, negative results identify model blind spots, and ADMET data guide improvements to molecular representations. This iterative process distinguishes contemporary AI-driven NP discovery from traditional high-throughput screening by enabling ongoing model refinement.

Notably, the transition from computational prediction to clinical translation remains incomplete for most AI-identified NPs. As illustrated in our case studies (Section 8), current examples range from Level C (computational hypotheses) to Level A (in vivo validation), yet none have progressed to clinical trials to date. This gap reflects the inherent challenges of NP drug development—including synthetic accessibility, formulation complexity, and pharmacokinetic optimization—as well as the relatively recent development of robust AI methodologies for NP discovery. Bridging this translational divide necessitates early incorporation of developability criteria, as discussed in Section 9, and the adoption of prospective validation frameworks, as outlined in Section 10. This review endeavors to equip researchers with both computational tools and the translational context required to navigate this comprehensive discovery-to-validation pathway effectively. Figure 1 offers a schematic overview of this integrated workflow, demonstrating how computational methods (absorption, distribution, metabolism, excretion, and toxicity (ADMET) optimization, ligand-based virtual screening (LBVS), structure-based virtual screening (SBVS), and network pharmacology) inform experimental validation through iterative active learning cycles.

## 2. Overcoming Pharmacokinetic Limitations Through AI-Driven Optimization

It is established that pharmacokinetic challenges have historically hindered the clinical adoption of various NPs despite their potential in preclinical models. For example, curcumin exhibits promising effects in preclinical studies; however, its systemic absorption is limited when taken orally due to poor intestinal uptake, significant first-pass metabolism, and rapid clearance. These limitations are linked to physicochemical properties common among NPs, such as high molecular weight, numerous hydrogen bond donors and acceptors, and complex stereochemistry [33,34,35]. Modern methods treat oral bioavailability as an optimizable property rather than an intrinsic limitation. Combining computational prediction of ADMET properties with formulation science and medicinal chemistry enables an iterative process that systematically addresses pharmacokinetic issues.

Computational ADMET platforms facilitate the rapid identification of developability issues before synthesis or extensive biological testing. Tools like SwissADME web server (SIB Swiss Institute of Bioinformatics; http://www.swissadme.ch, accessed on 20 December 2025) and ADMETlab 2.0 offer scalable evaluations of lipophilicity, aqueous solubility, gastrointestinal absorption potential, and cytochrome P450 interaction risk [36,37]. Advanced deep learning architectures tackle specific technical challenges of NPs. For instance, self-supervised pretraining and transfer learning address the limited availability of labeled bioactivity and ADMET data. In addition, 3D geometric models, including SE(3)- and E(3)-equivariant graph neural networks (GNNs) and transformers, effectively capture stereochemical and conformational features that 2D representations cannot fully encode [38,39,40]. ML approaches can improve ADMET predictions for NPs in several ways when training data adequately represent NP chemical space, though performance might vary substantially across endpoints. First, expanded training datasets now include NP-rich chemical libraries, and transfer learning from large unlabeled molecular datasets enhances generalization across SMILES, graph, and 3D representations. Second, Conformal prediction and Bayesian approaches identify compounds outside the applicability domain, flagging unreliable predictions rather than generating overconfident estimates [41,42,43]. Third, multitask architectures predict multiple ADMET endpoints simultaneously, while metabolite-aware pipelines incorporate biotransformation predictions [44]. Lastly, local ML engines enable rapid processing at a million-scale throughput with active learning, continuously refining models [45].

Moreover, physiologically based pharmacokinetic (PBPK) modeling combines drug physicochemical properties with anatomical and physiological parameters to predict drug concentrations in plasma and tissues over time [46]. These models guide dose determination, assess variability in pharmacokinetics, and evaluate potential risks of drug–drug interactions (DDI) [46]. Recently, hybrid methods have integrated ML with mechanistic PBPK models, utilizing structural data to estimate complex ADME parameters and enhance physiological modeling. This fusion accelerates testing hypotheses related to exposure, tissue distribution, and metabolism, while maintaining the mechanistic clarity required for regulatory purposes [47,48,49,50].

## 3. AI-Enabled Molecular Representation and Target Prediction

### 3.1. Representing NP Structural Complexity

NPs pose substantial constraints for computational modeling, owing to their unique bioactive scaffolds, which typically exhibit dense stereochemistry, macrocyclic constraints, glycosylation patterns, and a high heteroatom content. Therefore, canonical 2D structural representations, such as SMILES and molecular graphs, can capture bond structures but do not adequately capture 3D conformational or chiral geometries, which are essential for biological activity. Consequently, this compromises the accuracy of predicting geometry-dependent properties, such as membrane permeability, transporter interactions, and stereoselective binding [51].

GNNs represent molecules as feature-annotated graphs, where atoms are nodes and bonds are edges, each of which is associated with learned or engineered features, such as element type, valence, aromaticity, formal charge, and other chemoinformatic descriptors. While 2D graphs reflect local chemical environments, capturing 3D geometry requires conformer-based features or geometric architectures that retain symmetry. Most molecular GNNs use message-passing frameworks, in which node states are updated by aggregating information from neighbors over iterations. These are followed by readout steps that produce molecule-level embeddings. The number of message-passing steps sets the receptive field, with shallower networks often generalizing better than deeper ones [52,53,54]. In the scope of NPs, three-dimensional geometric representations offer essential advantages. E(3)- and SE(3) architectures—including SchNet, DimeNet, GemNet, and geometric transformers—enforce rotation/translation equivariance, yielding invariant scalar predictions after appropriate readout, enabling the encoding of conformational and stereochemical details, which are essential for NP analysis [52,55,56]. In addition, AttentiveFP is an example of a graph attention model for molecular property prediction, delivering strong performance and atom-level attention maps that support interpretability [57].

**Figure 2:**
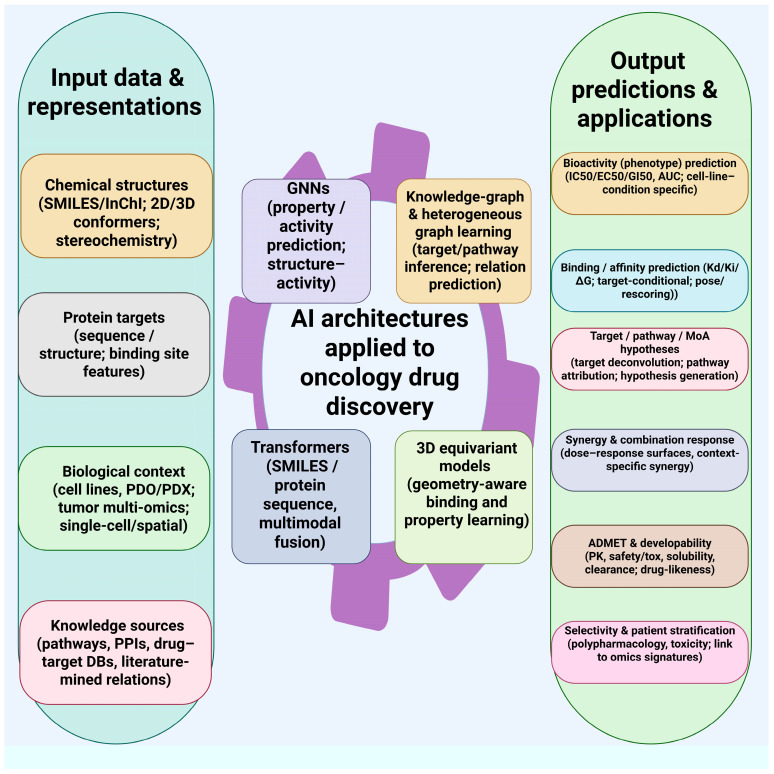
This schematic overview illustrates a multimodal AI pipeline designed for oncology discovery and optimization. It encodes chemical, target, biological context, and assay metadata into molecular and multimodal representations using graph-based, transformer-based, and generative methods, as well as knowledge graphs. The pipeline produces outputs such as phenotype-specific bioactivity, target-conditional binding and affinity, hypotheses on target pathways and mechanisms of action (MoA), combination-response predictions, and developability assessments, including ADMET and selectivity profiling. These predictions are continually refined through experimental validation and preclinical steps within an active-learning Design–Make–Test–Analyze (DMTA) cycle, enabling clinical prioritization and effective translation. Created with BioRender.com.

Recent advances in self-supervised pretraining for molecular representations have significantly improved the performance of property prediction tasks essential to drug discovery. GEO-BERT, a geometry-based bidirectional encoder, utilizes three-dimensional positional data—such as atom-atom, bond-bond, and atom-bond relationships—to more accurately characterize molecular structures [58]. Validation studies have identified two potent DYRK1A inhibitors (IC_50_ < 1 μM), underscoring their potential in kinase-targeted anticancer research. Furthermore, Multi MoleScale integrates graph contrastive learning with BERT-based sequence models, outperforming previous approaches on datasets involving breast cancer cell lines and ADMET prediction, all without relying on handcrafted features [59]. For NP discovery related to metastasis and chemoresistance, these pretrained molecular representations can improve transfer learning into chemical space with limited labeled data. Figure 2 illustrates how various AI workflows encode chemical, target, and biological data into molecular and multimodal representations. These workflows use different architectures, including graph-based, transformer-based, generative, and knowledge graph methods, to aggregate learned features into a final prediction.

### 3.2. CPI Prediction

In NP discovery workflows, GNNs facilitate the prediction of molecular properties and ADMET across diverse chemical libraries. Additionally, GNNs can model drug–target interactions (DTI) and binding affinities using ligand and protein data and can profile polypharmacology to predict activity across multiple targets. However, predicting binding kinetics is more challenging than predicting affinities due to limited training data [60,61,62]. For instance, transformer- and GNN-based frameworks for CPI enable target-focused screening of structurally diverse NPs by learning relationships between sequences, structures, and ligands that go beyond traditional docking heuristics [63,64]. Within this paradigm, Siamese encoder architectures with shared weights offer an elegant solution for comparative analysis: by embedding compound pairs into a unified latent space, these networks can model pairwise relationships such as structural similarity, binding profile comparisons, or activity cliff detection. This is especially advantageous in NP research, where structurally related analogs often exhibit divergent bioactivities, and where rapid prioritization of candidates against reference compounds is essential [65]. To predict drug synergy in combination, multimodal fusion methods combine drug representations with cellular context features, such as baseline gene expression, mutation profiles, and pathway activities, to produce cell line-specific predictions [66]. Multi-view molecular representation learning addresses the limitations of methods that rely on a single modality by integrating 1D, 2D, and 3D molecular data. MolMVC employs attention-guided augmentation and an adaptive multi-view contrastive loss to align molecular representations across these modalities [67,68]. The approach has shown improved results in predicting molecular properties, drug-target affinity, and cancer drug response—crucial areas for NP chemosensitization. These techniques could also boost NP activity predictions in resistant cancer cell lines by effectively capturing stereochemical and conformational features that distinguish active NP stereoisomers.

Several critical limitations warrant emphasis. While binding affinity prediction has achieved considerable success, modeling binding kinetics—encompassing parameters such as association rate (k_on_), dissociation rate (k_off_), and residence time—remains substantially less developed due to the scarcity of experimentally derived kinetic data. This represents a significant gap, as drug-target residence time is increasingly recognized as a key determinant of in vivo efficacy and duration of action. Additionally, benchmark datasets often contain systematic biases, including overrepresentation of specific ligand scaffolds or protein families, which can artificially inflate apparent model performance. To assess genuine predictive capability, rigorous evaluation protocols are essential, including label reversal experiments to detect target leakage, temporal splits that simulate prospective discovery scenarios, and scaffold-based partitioning to evaluate generalization beyond training chemotypes [69,70]. Over the past decade, the significance of drug-target residence time—the duration of the drug-target complex—has increasingly been recognized as a critical factor influencing in vivo pharmacological effects [71]. Unlike the binding affinity at equilibrium, residence time reflects the dynamic conformational changes in target molecules that affect how drugs bind and release. This is especially important for NPs targeting kinases involved in metastasis and transporters linked to resistance, where more extended target engagement might be necessary for sustained pathway suppression. For instance, longer residence times may be needed to inhibit EMT transcription factors and prevent mesenchymal program recovery. In contrast, brief inhibition of P-glycoprotein might not sustain sufficient intracellular drug levels. Currently, most AI models for compound–protein interaction prediction are trained on equilibrium endpoints like Kd, Ki, or IC50. In contrast, predicting binding kinetics such as k_on_, k_off_, and residence time is still developing, mainly due to the scarcity of standardized kinetic datasets. For instance, although BindingDB contains about 3.2 million binding data points in total, it offers only a limited number of measurements for k_on_ and k_off_. This demonstrates a significant imbalance between affinity data and kinetic data. Therefore, creating standardized, well-annotated experimental kinetic datasets—with consistent assay formats and metadata for techniques like SPR, BLI, and stopped-flow—is essential for advancing scalable kinetic ML, rather than being an afterthought. Meanwhile, physics-based methods such as enhanced-sampling molecular dynamics and weighted-ensemble approaches, along with hybrid ML/MD strategies, can offer complementary estimates of kinetic trends. However, they can be computationally intensive [72,73]. Incorporating kinetic parameters into AI-based NP screening methods is a crucial step in improving the success of translational oncology research. Several essential NPs exhibit binding kinetics that underscore the importance of residence time. For example, Paclitaxel binds to tubulin with a KD of about 2 μM under cooperative binding conditions with Tau and exhibits dissociation kinetics that enable prolonged microtubule stabilization, a property vital for its antimitotic activity [74]. Likewise, hemiasterlin and its synthetic analog HTI-286 demonstrate that NPs with similar equilibrium affinities (KD ~100 nM) can exhibit different kinetic profiles, which can affect their pharmacological effects [75]. These instances highlight that for NPs targeting processes related to metastasis—such as microtubule dynamics during cell migration or kinase signaling during EMT—binding kinetics can be as crucial as equilibrium affinity in therapeutic outcomes.

### 3.3. Addressing Transporter-Mediated Resistance

Transporter-mediated drug efflux, particularly through ABCB1/P-glycoprotein, plays a substantial role in chemoresistance [76,77,78]. ML and deep learning approaches have emerged as valuable tools for predicting P-gp substrate and inhibitor status. GNN-based approaches, such as AttentiveFP and ensemble models, now provide reliable predictions of P-gp substrate status. In large-scale NP screening, these models tend to deprioritize scaffolds that are likely to be rapidly effluxed in resistant tumor settings or highlight candidates for chemosensitization strategies [79]. Lim et al. showed that GCNN models enhanced with quantum chemical descriptors significantly improved P-gp efflux prediction versus models without augmentation [80]. Similarly, a multiclass classifier was developed for P-gp substrates, inhibitors, and inactive compounds. It uses counter-propagation artificial neural networks trained on 2512 compounds and achieved a non-error rate of 0.85 on the test set [81]. Prachayasittikul et al. also demonstrated effective classification of 2477 P-gp-interacting compounds using decision trees, artificial neural networks, and support vector machines, with Matthews’ correlation coefficient (MCC) values spanning from 0.665 to 1.0 for external validation [82].

In addition to substrate prediction, the BEAR method effectively identified and experimentally confirmed nine new dual P-gp/BCRP inhibitors, leveraging bioactivity-based deep learning. It surpasses other ML models in targeting the ABC transporter family [83]. These computational advances support ongoing research into NP-derived chemosensitizers. Ma et al. provided a thorough review of traditional Chinese medicine monomers and derivatives that act as P-gp-mediated MDR reversers, including those with both cytotoxic and P-gp inhibitory effects [84]. Among these, curcumin has demonstrated P-gp inhibitory activity in preclinical models, though its clinical significance is limited by poor systemic bioavailability [33]. In large-scale NP screening, these predictive models can identify scaffolds unlikely to be rapidly effluxed in resistant tumors and prioritize candidates for chemosensitization strategies.

## 4. Virtual Screening for NP Discovery

### 4.1. The Scale Challenge

Virtual screening enables the rapid computational prioritization of potentially bioactive molecules from vast chemical libraries, making it essential for the discovery of NPs. Contemporary resources, including COCONUT 2.0 (695,133 unique structures from 63 sources) [85] and LOTUS (approximately 750,000 structure-organism pairs) [86], provide curated NP structures with taxonomic annotations. Meanwhile, extended NP-like libraries include tens of millions of compounds. Due to the impracticality of experimental testing on such extensive collections, computational methods are required to prioritize candidates effectively. Beyond database curation, computational methods for prioritizing NP-like compounds rely on quantitative metrics that differentiate authentic NP scaffolds from synthetic molecules. The NP-likeness score, a Bayesian measure based on the frequency of fragment occurrences in NP versus synthetic collections, allows for a quick assessment of a virtual library’s “naturalness” [87]. This score effectively separates NPs from synthetic molecules. It has been used to focus compound libraries toward NP-like chemical space, assist virtual screening, and guide the design of building blocks for NP-like library synthesis. For targets related to metastasis and chemoresistance, NP-likeness scoring helps ensure that AI-generated analogs preserve key structural features associated with NP polypharmacology, while identifying compounds that may become more synthetic-like during optimization. Recent implementations have expanded NP-likeness scoring to evaluate NP-inspired combinatorial libraries, showing that virtual libraries based on NP scaffolds, such as anthraquinones and chalcones—both with known anticancer activity—can retain NP-likeness similar to that of FDA-approved drugs [88].

### 4.2. Structure-Based Virtual Screening

SBVS involves docking candidate ligands into target binding sites and assessing potential binding modes using three-dimensional structural data. It helps generate hypotheses about target interactions for compounds without bioactivity annotations and reveals clear patterns of protein–ligand interactions [89]. However, docking-based virtual screening typically achieves experimental hit rates of 1–10% when top-ranked compounds are tested, though rates vary substantially depending on target tractability, library composition, and the number of compounds experimentally evaluated [90,91]. To address this, modern SBVS workflows use receptor ensemble docking across multiple conformations, consensus scoring with various functions, and rank-based methods to reduce biases [92]. NPs require careful consideration of ligand protonation states, tautomers, stereochemistry, and conformations, as these factors significantly influence docking outcomes.

AI can enhance SBVS capabilities in several ways: for instance, AI can expand structural coverage, as exemplified by the substantial expansion in this field following AlphaFold2, which substantially expanded structural coverage for structure-based approaches [93]. However, predicted structures may exhibit conformational differences in binding sites relative to ligand-bound experimental structures, and a critical assessment of binding-pocket geometry is advisable before large-scale docking campaigns. Moreover, tools such as GNINA employ convolutional neural network-based scoring functions that improve docking accuracy. On redocking benchmarks, GNINA v1.0 increases top-1 pose accuracy (RMSD < 2 Å) from 58% to 73% compared to AutoDock [94]. In virtual screening, early enrichment factors are approximately 2-fold higher than those reported by Vina on DUD-E/LIT-PCBA benchmarks [95]. Though it should be noted that these datasets harbor known biases that may inflate apparent performance relative to prospective campaigns. DiffDock, as an example of generative docking and other related diffusion-based approaches, enables rapid pose generation with confidence metrics [96,97]. Finally, AI can accelerate virtual screening by using Deep Docking and other related model approaches, enabling billion-scale screening campaigns to proceed approximately 100-fold faster while retaining approximately 90% of top-scoring compounds at typical recall settings, with user-adjustable recall thresholds balancing speed against hit recovery [98].

### 4.3. Ligand-Based Virtual Screening

In contrast to SBVS, LBVS ranks compounds based on their chemical similarity to known active molecules. Typically, it involves selecting reference ligands, extracting molecular features such as fingerprints and pharmacophore descriptors, and then ranking candidates using similarity metrics or supervised classification methods. Results are validated through retrospective enrichment analysis and prospective testing [99]. Similar to the progress seen in SBVS, AI can enhance LBVS by replacing rigid similarity rules with learned molecular representations, thereby improving scaffold representations across a wide range of chemical libraries. Models such as graph attention, message-passing networks, and self-supervised encoders, including GROVER graph transformers and ChemBERTa SMILES transformers, enable fast, large-scale ranking. These models can be fine-tuned for targets related to resistance. Uncertainty-aware ranking enables the investigation of chemotypes outside the training domain rather than excluding them with strict cutoffs [57,100,101].

### 4.4. Hybrid Workflows for Metastasis and Chemoresistance

Combining LBVS and SBVS is an optimal virtual screening strategy, particularly when guided by phenotypic and genomic data, as it leverages the complementary strengths of both approaches to maximize lead identification and generate mechanistic hypotheses. In this hybrid paradigm, LBVS rapidly enriches NP chemotypes that align with known actives or influence resistance [102,103,104]. Subsequent SBVS generates plausible targets and interaction hypotheses for this shortlist, typically through ensemble docking and rescoring [105,106].

This comprehensive framework has demonstrated strong effectiveness in identifying NPs that target resistance-related signaling pathways. For example, gene expression-based virtual screening combined with network analysis identified withaferin A as an effective chemosensitizer that works synergistically with NOTCH1 inhibition in γ-secretase inhibitor-resistant T-cell acute lymphoblastic leukemia by inducing eIF2A phosphorylation, leading to translation inhibition [107]. Likewise, structure-based virtual screening of the ChEBI library found G3, a natural compound derivative that overcomes resistance to 5-fluorouracil and oxaliplatin in colorectal cancer by blocking XLF-mediated DNA repair [108]. In the context of metastasis, structure-based pharmacophore mapping and virtual screening of NP databases have revealed polypharmacological inhibitors like Cedeodarin, which concurrently target c-MET, EGFR, and VEGFR-2 receptor tyrosine kinases involved in tumor invasion and metastatic spread [109]. ML-enhanced hybrid methods have advanced this capability further, as demonstrated by random forest-based virtual screening of phytochemical libraries, which successfully discovered new KRAS(G12C) inhibitors that surpass approved drugs in binding affinity assessments [110]. These examples illustrate how hybrid virtual screening connects NPs hits with critical resistance mechanisms—including EMT transcriptional programs, CSC pathways, and drug efflux transporters—while providing structural rationale for observed phenotypic effects.

### 4.5. Fragment-Based Approaches Using NP Scaffolds

Fragment-like NPs are a strategically valuable subset for AI-driven discovery, as they are easier to synthesize and analyze compared to complex NP structures. Studies indicate that these fragments often retain the biological relevance of the original NPs while being more accessible for medicinal chemistry enhancements [111]. For instance, Tang et al. employed NMR-based fragment screening and AI-guided virtual screening to identify flavonoid NPs—myricetin, vitexin, and hyperoside—that serve as potent RNase L inhibitors with possible anticancer effects [112]. The structural similarity between these hits and bioactive NPs facilitated the rapid identification of more potent natural variants, illustrating how fragment screening can efficiently explore NP chemical diversity. To target metastasis and chemoresistance, breaking down validated NP hits using Murcko scaffold decomposition or similar methods can yield targeted fragment libraries that combine NP-based privileged structures with simpler molecules suitable for further optimization.

## 5. Network Pharmacology and Systems-Level Target Deconvolution

Network pharmacology offers a comprehensive approach to understanding mechanisms and identifying targets, extending beyond the traditional single-drug-one-target concept to investigate multi-target interactions within intricate disease networks. This is mainly achieved by integrating chemoinformatics, systems biology, and network analysis, which help in selecting the most promising targets and pathways for experimental validation [16,113,114]. Typical pipelines begin with compound–target interactions, followed by ligand-similarity analysis. The predicted targets are then compared with disease-related gene sets from sources such as GeneCards, DisGeNET, or OMIM. These standard targets are mapped onto protein–protein interaction (PPI) networks, commonly using STRING, to clarify their roles within larger disease networks. This process is followed by docking studies and pathway identification [115,116].

AI is advancing network pharmacology by enhancing the analysis of compound–target interactions. These AI-driven approaches construct networks using deep learning models that predict DTI and CPI. These models reveal complex relationships, manage extensive sets of targets, and enhance traditional methods [117,118,119]. Various neural network architectures have proven effective in predicting DTI. For instance, convolutional neural networks (CNNs) can identify spatial and molecular features crucial for these interactions. Models like CSatDTA utilize convolutional self-attention mechanisms to capture long-range interactions within molecular sequences, enabling them to outperform earlier sequence-based approaches [120]. GNNs operate directly on molecular graphs, offering an intuitive way to learn the complex topological and geometric properties of drug-like molecules. These models are popular in molecular tasks such as property prediction, virtual screening, and synthesis planning [121]. Deep Collaborative Filtering models like DCFME employ multiple representation learning techniques to extract features from heterogeneous networks. They simulate DTI from different perspectives, leading to significant performance gains on sparse datasets [122]. Transformer-based models improve prediction accuracy by capturing complex dependencies in biological sequences through attention mechanisms, capturing noncovalent interaction patterns relevant to binding [123].

A significant benefit of deep learning is its ability to predict targets at scale. These techniques overcome the limitations of traditional experiments and offer insights beyond those provided by pharmacophore and docking methods [124]. Additionally, they include uncertainty quantification, which provides reliability estimates essential to network pharmacology. This is particularly important because out-of-domain errors can affect subsequent analyses. Conventional deep models often show overconfidence, especially with out-of-domain (OOD) data, risking costly mistakes [125]. This is especially critical for NPs, which usually have diverse structures, making OOD detection and error propagation challenging. Bayesian methods, including deep ensembles, Monte Carlo dropout, Laplace approximation, and Bayes by Backprop, provide probabilistic estimates of uncertainty. Research indicates that Bayes by Backprop often improves calibration and more reliable uncertainty estimates without additional calibration [126]. AI further improves network integration by utilizing knowledge graphs and graph ML. These tools generate embeddings that facilitate link prediction and hypothesis ranking across various evidence types, including chemistry, targets, protein interactions, pathways, phenotypes, and clinical outcomes. This process enables quicker, data-driven prioritization of multi-target mechanisms, helping to identify biases and coverage gaps [127,128]. Knowledge graphs integrating chemical, biological, and clinical data provide structured methods for generating target hypotheses. SKiM (Serial KinderMiner) uses literature-based discovery alongside transformer models to find A-B-C relationships, connecting compounds to disease mechanisms through intermediate concepts [129]. In NP discovery, these methods can systematically examine links between NP scaffolds and resistance mechanisms reported across various sources, potentially revealing repurposing opportunities for existing NPs to combat chemoresistance.

Another effective alternative is evidential deep learning (EDL), which quantifies uncertainty through learned evidence. The EviDTI model, utilizing EDL, combines drug 2D topological graphs, 3D structures, and target sequences to guide experimental validation toward higher-confidence predictions [118]. Hybrid Bayesian and evidential approaches, like the Ensemble of Evidential models, often achieve the best performance by providing insights into both accuracy and uncertainty at lower computational costs [130]. Conformal Prediction Methods offer well-calibrated intervals with theoretical guarantees. For example, conformalized fusion regression (CFR) merges GNNs with mean-quantile regression for reliable ADMET predictions [131]. Uncertainty quantification methods, such as Bayesian approaches, deep ensembles, and conformal prediction, help identify predictions of questionable reliability. This is especially useful when screening structurally diverse NPs. However, most of these methods have been calibrated on synthetic drug-like compounds and have not been thoroughly validated for NP chemical space. Since NPs often appear outside typical training distributions, their uncertainty estimates may be inaccurate precisely where they are most crucial. Therefore, researchers should view computational confidence intervals as tools for generating hypotheses rather than definitive answers, and should rely on experimental validation for both high-confidence and high-uncertainty predictions. The UAMRL framework uses a dual-stream encoder and models uncertainty with the normal-inverse-gamma distribution to evaluate the reliability of diverse data [132]. Similarly, models for Traditional Chinese Medicine, like MMFi-DPBML, incorporate multiple molecular features to ensure robust generalization [133].

**Figure 3:**
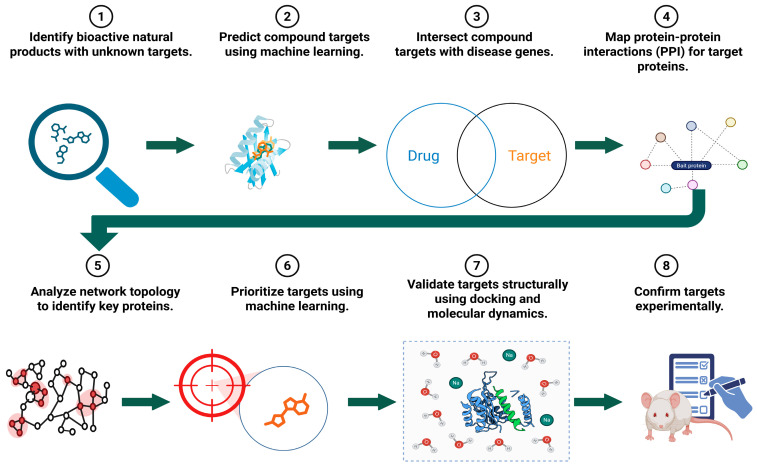
An ML-driven workflow for discovering and verifying bioactive NPs starts with ML predicting possible protein targets for an NP with an unknown molecular target. These predictions are matched with disease-related genes from GeneCards and DisGeNET to find clinically relevant candidates. Next, the candidates are expanded and analyzed within PPI networks in STRING, where features like hubs and bridges help identify potential targets and pathways. These network features, along with disease relevance, inform an ML model that ranks the targets. The top candidates are validated through molecular docking and molecular dynamics simulations to assess binding stability. Finally, biochemical and cellular assays confirm the results. Created with BioRender.com.

These capabilities are essential for metastasis and chemoresistance, which involve complex systems rather than just single genes. AI-driven network pharmacology models the complexities of biology by integrating tumor-intrinsic factors with the microenvironment. Network analysis identifies key nodes—such as highly connected hubs or bridging elements between pathways—that could be targets for intervention. However, importance in network topology does not automatically imply biological causality or druggability; highly connected nodes may be involved in essential cellular functions rather than disease-specific vulnerabilities, and bridge positions may not be crucial for disease progression. Consequently, hypotheses from network analysis require experimental validation to confirm genuine causal and druggable targets, separating actual targets from network artifacts. When confirmed, this approach can strengthen polypharmacology strategies, in which targeting multiple sites is viewed as a therapeutic feature rather than an off-target liability (Figure 3) [134,135]. Overall, AI has greatly improved network pharmacology by enhancing the modeling of compound–target interactions with deep learning. These techniques reveal complex relationships, manage large sets of targets, and improve traditional experimental methods. Future developments in geometric deep learning, self-supervised pretraining, and uncertainty modeling are expected to further advance the role of network pharmacology in drug discovery.

## 6. Decision Framework for AI Method Selection

**Figure 4:**
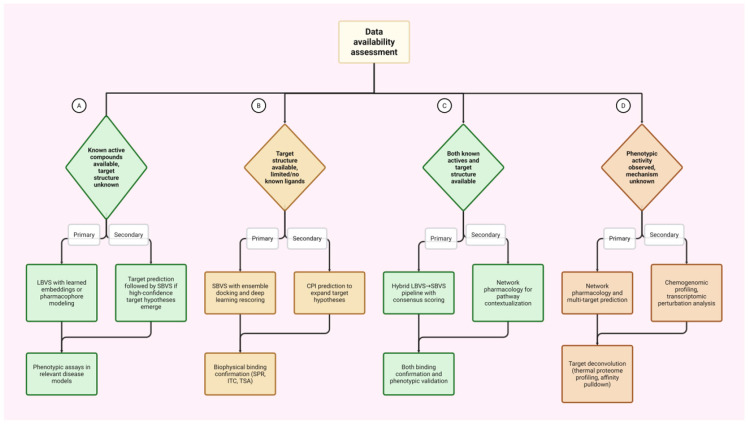
Decision Framework for AI Method Selection in NP–Focused Drug Discovery. The workflow selects computational approaches based on (i) data availability and target characterization (Scenarios (**A**–**D**)). Created with BioRender.com.

Based on our literature review, we propose an operational framework for selecting appropriate AI methodologies given available data, target characterization, and validation resources. Rather than applying computational approaches indiscriminately, researchers should align their methodological choices with the specific constraints and opportunities of their discovery context. Choosing the right computational strategy mainly depends on the type of data available. If active compounds are known but the target structure is unknown (Figure 4A), LBVS using learned embeddings or pharmacophore modeling is the most suitable initial method. If high-confidence target hypotheses emerge, target prediction algorithms can then guide structure-based screening, with phenotypic assays in relevant disease models serving as validation. Conversely, when target structural data is available—either through experimental methods or AlphaFold predictions—but few or no ligands are known (Figure 4B), SBVS with ensemble docking and deep learning rescoring is preferred. In this case, CPI prediction can help generate new target hypotheses, and biophysical validation techniques, such as SPR, ITC, or CETSA, are critical for confirmation. When data on both known actives and target structures are accessible (Figure 4C), hybrid strategies that combine ligand-based filtering with structure-based refinement and consensus scoring across diverse approaches are advantageous. Network pharmacology offers valuable pathway context, and validation should include both binding confirmation and phenotypic testing. Finally, if phenotypic activity is observed but the mechanism is not precisely defined (Figure 4D), network pharmacology and multi-target prediction serve as effective starting points. Chemogenomic profiling and transcriptomic perturbation analysis can support these efforts, with target deconvolution via thermal proteome profiling (TPP) or affinity pulldown experiments being essential validation steps.

Furthermore, computational resource needs grow significantly with library size, requiring strategic adjustments in methodology. For libraries with fewer than 10,000 compounds, standard molecular docking remains manageable, and accuracy should be prioritized over speed. Libraries containing between 10,000 and 1,000,000 compounds benefit from deep learning scoring functions and surrogate models, which offer good accuracy with reduced computational effort. When screening libraries larger than 1,000,000 compounds, multi-stage filtering becomes crucial, typically starting with ADMET filtering, followed by ligand-based enrichment, and finally focused structure-based evaluation of the selected subsets. Frameworks like Deep Docking [98], which can reduce computational costs by approximately 100 times while still capturing most top-scoring compounds, are beneficial at this scale. Crucially, synthetic accessibility must be integrated as a primary filter from the beginning of any AI-driven NP screening process, rather than being considered later. If a computationally prioritized NP cannot be produced in adequate quantities—whether through extraction, semi-synthesis, or total synthesis—the entire discovery process stalls at the translational stage, regardless of its predicted bioactivity or target engagement. Therefore, we suggest incorporating a synthetic accessibility score (SAScore) that combines fragment contributions from known chemistry with penalties for molecular complexity as a key part of the initial filtering. This score provides a quick, validated computational estimate of synthesis feasibility and should be applied to all AI-prioritized hits [136]. It is also important to distinguish between biosynthetic route prediction tools like BioNavi-NP (http://biopathnavi.qmclab.com/job.html, accessed on 20 December 2025), which model enzymatic pathways and biogenetic building blocks, and chemical synthesis planning tools such as ASKCOS (https://askcos.mit.edu/, accessed on 20 December 2025) [137] and AiZynthFinder v4.4.1 [138], which evaluate laboratory synthesis routes. For NP discovery aimed at translation, both perspectives are essential: biosynthetic analysis informs fermentation and heterologous expression strategies, while retrosynthesis tools assess the practicality of total or semi-synthesis. Compounds that perform poorly in both biosynthetic sourcing and synthetic accessibility should be deprioritized or flagged for analog development, regardless of their predicted biological activity.

Before deploying any AI method, researchers should carefully evaluate several key factors affecting prediction reliability. Initially, assessing the overlap between training data and the query library or target protein family is crucial. Significant overlap can inflate performance metrics and may not indicate true predictive power. Next, the applicability domain must be considered, as query compounds outside the chemical space of the training data—familiar with NPs, which are trained mainly on synthetic compounds—can lead to unreliable predictions. Additionally, the relevance of benchmark datasets such as MoleculeNet and DUD-E must be scrutinized, as they contain known biases that may not accurately reflect real discovery scenarios. Finally, distinguishing between prospective and retrospective validation is essential: retrospective tests on curated datasets often overestimate real-world hit rates; therefore, researchers should adjust their expectations accordingly. Additionally, the data splitting strategy significantly influences the accuracy of performance estimates for AI models in NP discovery. Randomly splitting data often overestimates performance because structurally similar compounds appear in both training and test sets. To enhance reliability, using scaffold-based splits is recommended as a standard approach, in which compounds sharing a Murcko scaffold are assigned exclusively to either the training or test set. Temporal splits, which separate data by publication date, better simulate real-world discovery processes and usually yield more cautious, realistic performance estimates [139]. Validating models with independent datasets from different sources remains the best practice before moving to experimental testing. For applications involving metastasis and chemoresistance—where scaffold novelty is often therapeutically critical—models trained only on random splits may perform poorly across diverse NP libraries. We advise reporting metrics across various split types to evaluate model generalizability transparently. It is essential to recognize that scenarios (A–D) assume some prior knowledge, such as existing actives or target structures. However, discovering NP for new resistance mechanisms often faces a “doubly data-poor” situation where reliable compounds and well-characterized targets are absent. This common scenario in early-stage exploration of understudied resistance pathways requires a different approach. We recommend an integrated, phenotype-first workflow: (i) begin with phenotypic screening in relevant models (like resistant cell lines, 3D spheroids, or patient-derived organoids) to identify active NPs or fractions against resistance, using assays such as differential cytotoxicity, invasion/migration, or CSC-targeting readouts; (ii) perform chemogenomic profiling—including transcriptomic signatures (e.g., CMap/LINCS), cell painting profiling, or TPP—to generate hypotheses about targets and pathways based on phenotypic hits [140,141,142,143]. (iii) use these hypotheses to train AI models via transfer learning from related target families or phenotypic endpoints, employing self-supervised molecular representations that do not require task-specific labels; and (iv) improve target hypotheses iteratively through active learning cycles, where each experiment expands the training set and refines the search space.

## 7. Experimental Validation and Active Learning

The computational identification of NPs targeting metastasis and chemoresistance pathways relies fundamentally on rigorous experimental validation. Predictions generated through virtual screening, network pharmacology, and CPI models represent hypotheses that must be confirmed in appropriate biological systems before therapeutic potential can be meaningfully assessed. This integration of computation and experimentation is particularly essential for NPs, which frequently exhibit structural diversity and polypharmacological profiles that challenge conventional single-target validation paradigms. Moreover, compounds identified through AI-based screening need to be validated across a range of concentrations to distinguish target-specific effects from those caused by overall cytotoxicity. Counter-screens using parental or isogenic target-knockout (KO) cell lines help distinguish on-target from off-target effects.

Furthermore, an essential but often overlooked step in NP hit validation involves spotting pan-assay interference compounds (PAINS). These are substructural motifs that commonly cause false positives in various biochemical assays due to nonspecific actions such as aggregation, redox cycling, metal chelation, and covalent reactivity [144]. Several NP scaffolds include recognized PAINS motifs such as quinones, catechols, and hydroxyphenyl groups. For NPs identified via AI-based screening aimed at metastasis or chemoresistance, applying PAINS filtering early in the validation process is essential to differentiate true target engagement from assay artifacts. Notably, compounds with these substructures are increasingly reported as potential drug candidates in the literature, which can confound the evidence for NP bioactivity claims. Most ADMET prediction platforms now incorporate computational PAINS filters, which should be routinely used on AI-prioritized NP hits before investing in resource-intensive validation experiments [144].

Predicted molecular interactions need confirmation via orthogonal biophysical methods that provide complementary information on binding properties. SPR measures binding kinetics in real time, determining association and dissociation rate constants and equilibrium dissociation constants, which quantify binding affinity [145]. ITC characterizes the thermodynamics of binding, measuring the enthalpy and entropy contributions to the binding free energy, thereby providing mechanistic insight into molecular recognition forces [146]. These cell-free approaches should be complemented by methods that confirm target engagement in cells, where the complex environment may influence behavior. The CETSA evaluates ligand-induced thermal stabilization of target proteins in cells or cell lysates, thereby demonstrating interactions under physiological conditions [147]. When the target remains uncertain despite predictions, TPP provides an unbiased method for identifying proteins with altered thermal stability following compound treatment, revealing both expected and unexpected interactions [148].

Functional assays should align with the therapeutic hypothesis, ensuring that the activities relate to the clinical goal. For compounds that inhibit metastasis, functional validation should include Wound healing, Transwell migration, Matrigel invasion, and Sphere formation assays should be used [149,150]. To assess chemoresistance, researchers can use cytotoxicity profiling in sensitive and resistant cell lines and assess shifts in inhibitory concentrations. Translation from cellular systems to therapeutic applications requires validation in physiologically relevant preclinical models that recapitulate key features of human disease. Patient-derived organoids are valuable, as they maintain the genetic heterogeneity and treatment response profiles of parental tumors while enabling moderate-throughput compound evaluation in 3D cultures that better mimic in vivo tumor architecture than monolayer cultures. Xenograft models using immunodeficient mice with human tumor cell lines or tissues enable in vivo efficacy, pharmacokinetic-pharmacodynamic (PK-PD), and preliminary toxicity assessment [151].

## 8. Case Studies: AI-Enabled NP Discovery for Metastasis and Chemoresistance with Mechanistic Integration

AI-driven NP discovery becomes biologically actionable when its predictions are mechanistically linked to pathway-level programs that clarify clinically relevant phenotypes, such as metastasis and chemoresistance. This connection is usually made through network pharmacology, which maps NP components to potential targets and modules like EMT, stem-like states, DNA repair, metabolic stress, and tumor–microenvironment interactions. However, network pharmacology is most potent when supported by additional mechanistic evidence, such as perturbation transcriptomics (e.g., CMap/LINCS signature matching), chemical-genomics or target fishing, ML-based phenotypic profiling with mechanism inference (e.g., image-based Cell Painting), knowledge graphs, causal or systems pharmacology models for multi-scale reasoning, and direct target validation methods like CETSA, TPP, and chemo-proteomics. These approaches collectively deepen mechanistic understanding, especially in deconvoluting polypharmacology, identifying responder subtypes, and predicting or reducing toxicity. AI and network pharmacology studies exemplify this strategy; some have progressed to experimental testing in colorectal cancer [152,153], while others are mostly computational, highlighting ongoing validation challenges [154]. To standardize the evidence linking an AI-prioritized NP to a mechanism (target-to-pathway) and a phenotype (e.g., metastasis or chemoresistance), we assign a score from 0 to 5 (Table 1). A score of 0 indicates in silico evidence only; 1 reflects in vitro phenotype data; 2 combines phenotype evidence with pathway readouts (like EMT markers, cell death pathways, immune/TME assays); 3 indicates in vivo efficacy, including effects on metastasis or in combination therapy; 4 signifies direct target engagement supported by biophysical or chemical biology methods (e.g., SPR, CETSA, chemoproteomics) along with pathway changes; and 5 involves physiologically relevant models (like organoids, orthotopic, or PDX models) showing target–pathway concordance. For practical purposes, Level C corresponds to scores 0–1, Level B to 2–3, and Level A to 4–5. When analyzing these case studies, it is essential to clarify AI’s specific contributions to each discovery. AI-powered research on NPs can assist at various stages: (i) identifying new active compounds from extensive, previously unexplored libraries; (ii) target deconvolution, whereby AI determines the molecular target or mechanism of action for known bioactive NPs; and (iii) providing mechanistic insights, where AI offers contextual pathway information and prioritizes hypotheses for testing. In the Level A cases discussed here, AI predominantly facilitated stages (ii) and (iii), rather than (i), as exemplified by the cases of Polyphyllins and Amentoflavone.

### 8.1. Transformer CPI Screening Identifies CD133-Targeting NP Polyphyllins

Transformer CPI Screening Identifies CD133 (PROM1) as a cell-surface glycoprotein marker for cancer stem cells across various tumor types. CD133-positive cells tend to have greater metastatic potential and are more resistant to therapy, making them a promising target for therapies aimed at eliminating tumor-initiating cells [155]. Hou et al. used TransformerCPI, a deep learning tool for predicting CPI, to screen a library of Chinese herb compounds for CD133 binders [156]. This AI-driven approach enabled the rapid prioritization of an extensive collection of NPs without requiring prior target-specific bioactivity data [156].

The screening identified two steroidal saponins—Polyphyllin V (PP10) and Polyphyllin H (PP24)—that have distinct downstream effects after CD133 engagement. Polyphyllin V mainly inhibits PI3K–AKT signaling and is linked to increased ROS, disrupted mitophagy/autophagy flux (mitophagy blockage), and GSDMD-related pyroptosis. Meanwhile, Polyphyllin H suppresses Wnt/β-catenin signaling, leading to apoptosis. Notably, the AI workflow focused on CD133 as the target and identified PP10/PP24 as potential binders; this was supported by docking studies and confirmed experimentally by SPR and CETSA, with dissociation constants (KD) of 5.4 × 10^−5^ M for PP10 and 1.0 × 10^−5^ M for PP24. Functional dependence was further validated by decreased sensitivity following CD133 knockdown. Mechanistically, PP24 reduced levels of pathway components, such as β-catenin, and lowered nuclear LEF1 and TCF1/TCF7, with apoptosis confirmed by Annexin V staining and changes including decreased Bcl-2 and increased Bax. Both compounds inhibited colorectal cancer cell migration and invasion in vitro, as shown by wound-healing and Transwell assays, and suppressed tumor growth in subcutaneous cell-derived xenografts with minimal toxicity. Their broader activity was supported by tests on patient-derived organoids and PDOX models across various tumor types, including colorectal, lung, and liver cancers, showing reduced proliferation markers, such as Ki67, and pathway activity in treated tumors. Overall, this work exemplifies how AI-backed predictions can be validated biologically, linking computational CPI predictions to actual target engagement and mechanisms, with demonstrated effectiveness in cell cultures, organoids, and xenograft models [156].

### 8.2. Network Pharmacology Identifies Amentoflavone as a TGFBR2 Inhibitor

TGF-β receptor signaling, primarily through TGFBR1 and TGFBR2, acts as a key upstream pathway in inducing EMT. Both EMT and partial-EMT processes are essential for invasion and the spread of metastases in advanced cancers, highlighting the pathway’s potential for targeted therapy [157]. Liu et al. [157] integrated network pharmacology with supervised ML to identify amentoflavone (AMF) targets in NSCLC and develop a core diagnostic gene signature. They linked computational predictions to metastasis-related functional tests and in vivo validation. Using NSCLC genes from MalaCards and PharmMapper-predicted AMF targets, they identified overlapping effectors, highlighting TGFBR2 as a key hub in the PPI network. A 13-gene signature selected by LASSO was modeled using XGBoost, achieving AUCs of 0.951 in internal validation and 0.814 in external validation. SHAP analysis identified TGFBR2 as a key risk factor, supporting a mechanistic hypothesis. Structural docking of the 13 proteins showed binding affinities from −12.3 to −7.7 kcal/mol, with strong AMF binding predicted for TGFBR2 (−11.1 kcal/mol), involving hydrogen bonds with HIS328/HIS340 and π–π stacking with PHE327. Authors treat docking as evidence of feasibility, not definitive binding. A 100 ns MD simulation confirmed complex stability, with backbone RMSD stabilizing around 1.45 Å after ~20 ns, maintained by 2–4 hydrogen bonds. Functionally, AMF reduced NSCLC cell migration in NCI-H1975 and NCI-H838 cells, using Transwell and wound-healing assays (0–10 μM, 24 h; mitomycin C minimized proliferation effects). Immunoblotting showed reversal of EMT: decreased N-cadherin, vimentin, and Slug, along with increased E-cadherin. TGF-β/Smad pathway activity was also reduced, as evidenced by lower TGFBR2 levels and decreased Smad2/3 phosphorylation. In vivo, a murine model of pulmonary metastasis with 4 × 10^6^ NCI-H1975 cells showed that daily intraperitoneal AMF (15 or 30 mg/kg, starting day 7 for 40 days) significantly decreased lung metastases, approaching the efficacy of cisplatin, with little impact on body weight. Overall, the study illustrates a workflow combining AI/ML, network pharmacology, structural modeling, pathway analysis, and in vivo validation, and also suggests AMF’s polypharmacology potential due to its favorable docking with multiple targets, including the metalloproteinases MMP2, MMP12, and MMP13 [157].

### 8.3. Deep Learning Virtual Screening Identifies Forsythoside A as an LOXL2 Inhibitor

Lysyl oxidase-like 2 (LOXL2) contributes to extracellular matrix remodeling via collagen crosslinking and has been implicated in EMT-associated transcriptional programs and pro-metastatic phenotypes in multiple tumor models. Consistent with this, elevated LOXL2 has been associated with invasion and metastasis across several cancer types, motivating continued development of LOXL2-directed strategies, although clinical efficacy remains unproven [158]. Jia et al. [159] utilized DeepVS, a graph-convolutional deep learning virtual screening tool, as their initial screening approach. They combined this with traditional docking methods and subsequent MM-PBSA analysis to find LOXL2 inhibitors within libraries of Traditional Chinese Medicine and NPs, highlighting Forsythoside A [159]. In CT26 colorectal cancer cells, Forsythoside A suppressed cell proliferation and decreased migration in scratch assays. The compound also increased apoptosis and lowered LOXL2 protein levels, confirmed by Western blot and ELISA, supporting the predicted target engagement from the computational analysis.

### 8.4. ML-Assisted Network Pharmacology for Identifying Potential Cisplatin Resistance Pathways

Cisplatin resistance in ovarian cancer involves complex mechanisms, including the rewiring of the DNA damage response (DDR), evasion of apoptosis, alteration of redox metabolism, increased efflux transporter activity, adaptation of cell-cycle checkpoints, and modification of the microenvironment [160]. This complexity makes it suitable for systems-level analysis and multi-target approaches. Zhong et al. [161] used an ML-assisted network pharmacology pipeline for *Stephania tetrandra*. Resistance-related genes were gathered from various sources and intersected with predicted targets of components. A PPI network was built, and four ML algorithms (random forest, SVM, generalized linear model, XGBoost) were trained with cross-validation to identify key targets. Docking, MD simulations, and ADMET analyses were then performed [161]. The ML layer highlighted core targets in PI3K-AKT, cell-cycle regulation, p53 signaling, and platinum resistance pathways. Docking and MD studies focused on specific constituent-target complexes for further research. This study represents computational hypothesis generation only; no experimental validation has been reported. It should not be interpreted as a validated discovery, but rather as a framework for hypothesis prioritization that requires experimental confirmation.

### 8.5. AI-Guided Target Identification in Apoptosis and Necroptosis Pathways

The Bupi Yichang Formula (BPYCF) study in colon cancer employs a systematic workflow combining network pharmacology with ML-guided prioritization, molecular docking, and laboratory validation. The study intersected BPYCF-related and colon cancer-related targets with differentially expressed genes (DEGs). A PPI network built with STRING facilitated the screening of key candidates using CytoHubba and MCODE, leading to the development of a random forest diagnostic model to pinpoint critical targets. The study identifies five primary targets, four of which consistently bind to the active compound naringenin. This was confirmed by HPLC–MS analysis in BPYCF. In vitro experiments demonstrated BPYCF’s ability to reduce colon cancer cell viability, lower NOS3 levels, and elevate the expression of CASP8, RIPK3, and TNFRSF10B. Overall, the findings suggest a coordinated regulation involving death-receptor/apoptosis pathways (CASP8, TNFRSF10B) and necroptosis signaling (RIPK3), offering pathway-based hypotheses relevant to resistance mechanisms, although the study did not directly assess chemoresistance phenotypes [152].

### 8.6. ML for Prognostic Gene Identification and Chemotherapy Toxicity Prediction

Huangqin Houpo decoction (HQHPD) in colorectal cancer (CRC) provides a translational example of ML-guided prognostic modeling combined with network toxicology and pharmacology to address chemotherapy-related adverse effects. Active components of HQHPD were identified via HPLC–MS, and their targets were gathered from multiple databases and integrated with transcriptomic data from TCGA and GEO. Using LASSO-Cox and multivariable Cox regression, an 11-gene prognostic signature was developed. GSEA and immune-function/infiltration analyses revealed that the high-risk group is enriched with oncogenic pathways and shows suppressed immune activity. In vivo, HQHPD reduced tumor growth in a dose-dependent manner, and flow cytometry showed decreased regulatory T cells (Tregs), indicating an immunomodulatory effect on the tumor microenvironment. Regarding chemotherapy toxicities, network pharmacology implicated PI3K/Akt signaling (including TP53, AKT1, SRC) in diarrhea and myelosuppression caused by 5-FU/irinotecan, with molecular docking confirming potential binding of HQHPD components to these targets. Lastly, 16S rRNA sequencing linked the reduction in diarrhea to changes in gut microbiota, such as increased Lachnospiraceae_NK4A136 and decreased Salmonella/Shewanella, suggesting a mechanistic basis for enhanced tolerability [153].

### 8.7. Single-Cell RNA Sequencing Integration with ML for Autophagy Targeting

Zhebeimu (ZBM; bulb of *Fritillaria thunbergii* Miq., Liliaceae) in TNBC demonstrates a comprehensive computational approach that combines network pharmacology, ML, and single-cell RNA sequencing, all explicitly described as in silico. The researchers identified 42 bioactive components of ZBM and 148 potential TNBC targets, then used ML to focus on five autophagy-related genes, with CXCR4 chosen as the key autophagy target for further analysis. Molecular docking and dynamics simulations indicated a strong binding between hapepunine and CXCR4. At the same time, single-cell subtype analysis revealed that basal-like (BL) TNBC might be especially responsive to ZBM compounds—supporting a hypothesis of subtype-specific effects rather than a general TNBC impact. The study also integrated network toxicology to predict possible hepatic and renal toxicity risks, which were computationally addressed by structurally modifying hapepunine derivatives. Overall, the research combines network pharmacology, ML, single-cell analysis, docking, dynamics, and toxicology prediction into a unified mechanistic framework. However, it does not include experimental in vitro or in vivo validation of target engagement or pathway causality, making it primarily a predictive study [154]. 

**Table 1:** AI-enabled NP case studies linking computational prioritization of target/pathway mechanisms to metastasis/chemoresistance-relevant phenotypes. Strength-of-evidence score (0–5).

Natural Product/Formula	AI/Computational Method	Primary Target(s) (Nominated/Tested)	Secondary Targets/Nodes (Reported/Predicted)	Pathway Readouts (Examples)	Metastasis/Chemoresistance Phenotypes	Model System(s)	Strength Score	Reference
Polyphyllin V (PP10)	TransformerCPI → docking; target engagement: SPR + CETSA	CD133 (PROM1)	Downstream network divergence emphasized (CD133-centered)	↓PI3K–AKT; mitophagy inhibition/blockade; pyroptosis program	↓migration/invasion; anti-tumor efficacy across models	Cancer cell lines; patient-derived organoids; animal models	5 (Level A)	[156]
Polyphyllin H (PP24)	TransformerCPI → docking; target engagement: SPR + CETSA	CD133 (PROM1)	Downstream network divergence emphasized (CD133-centered)	↓Wnt/β-catenin; apoptosis program	↓migration/invasion; anti-tumor efficacy across models	Cancer cell lines; patient-derived organoids; animal models	5 (Level A)	[156]
Amentoflavone (AMF)	Integrative network pharmacology + ML (gene signature/hub nomination) → docking + 100 ns MD; experimental validation	TGFBR2 (hub nominated)	Multi-node signature/module (PPI + ML-selected genes)	↓TGFBR2; ↓p-Smad2/3; EMT reversal markers (↑E-cadherin; ↓N-cadherin/vimentin/Slug)	↓migration/invasion; ↓pulmonary metastatic burden	NSCLC lines (e.g., NCI-H1975/NCI-H838); tail-vein lung metastasis model	3 (Level B)	[157]
Forsythoside A	DeepVS (GCN deep learning VS) → docking + MM-PBSA; target/pathway assays	LOXL2	ECM/EMT-linked network context	↓LOXL2 protein; apoptosis markers; migration suppression (consistent with LOXL2/ECM remodeling axis)	Anti-migratory phenotype (metastasis-relevant)	CT26 cells (and study-specific validation set); in vitro assays	2 (Level B)	[159]
*Stephania tetrandra* (cisplatin-resistant OC study)	Network pharmacology + ML (RF/SVM/GLM/XGBoost) + docking/MD + ADMET (hypothesis generation)	Multiple (prioritized hubs)	Enriched modules: PI3K–AKT, p53 signaling, cell-cycle, platinum-resistance pathways	Computational pathway enrichment/docking plausibility only	Chemoresistance context modeled (cisplatin resistance) without experimental confirmation	In silico (TCGA/GEO-style transcriptomic integration + databases)	0 (Level C)	[161]
Bupi Yichang Formula (BPYCF; naringenin-centered)	Network pharmacology → PPI (STRING; CytoHubba/MCODE) + random forest prioritization → docking + HPLC–MS + in vitro validation	NOS3, CASP8, RIPK3, TNFRSF10B (prioritized)	46 intersected candidates (formula targets ∩ disease genes ∩ DEGs)	↓NOS3; ↑CASP8/↑RIPK3/↑TNFRSF10B; viability suppression	Resistance-relevant death program reactivation (apoptosis/necroptosis); chemoresistance phenotype not directly tested	Colon cancer cell models (in vitro)	2 (Level B)	[152]
Huangqin Houpo decoction (HQHPD)	Network pharmacology + ML prognostic modeling (LASSO-Cox + multivariable Cox) + network toxicology; docking; in vivo + immune + microbiome assays	Prognostic gene set (11-gene signature); toxicity hubs in PI3K/Akt context (TP53/AKT1/SRC)	Immune/TME axes; microbiome remodeling as a tolerability mechanism	GSEA high-risk oncogenic enrichment; immune suppression signatures; ↓Tregs (flow cytometry); 16S microbiome shifts	Anti-tumor efficacy; toxicity-mitigation (chemo diarrhea/myelosuppression mechanisms)	In vivo tumor models; immunophenotyping; 16S rRNA sequencing	3 (Level B)	[153]
*Fritillaria thunbergii* Miq. (ZBM; hapepunine-centered)	Network pharmacology + ML + scRNA-seq integration; docking/MD; network toxicology + derivative optimization	CXCR4 (autophagy-associated target selected)	Autophagy-linked genes; subtype markers (basal-like TNBC)	In silico docking/MD; scRNA-seq subtype responsiveness hypothesis; predicted hepato/renal toxicity	Patient stratification hypothesis (BL TNBC); safety-liability prediction; no wet-lab causal validation	In silico + scRNA-seq analysis	0 (Level C)	[154]

“→” denotes a stepwise workflow link; “↓/↑” indicates decreases/increases in the stated readout (expression, phosphorylation, or pathway activity as specified); “∩” denotes set intersection (overlap) between gene sets.

## 9. Clinical and Translational Considerations for AI-Prioritized NP

AI-enabled NP discovery is only truly valuable in clinical settings when combined with (i) patient selection and PD monitoring, (ii) exposure-controlled formulation and PK/PD rationale, and (iii) manufacturing and quality systems that meet regulatory standards for identity, batch consistency, impurities, and stability. These elements are especially important for botanicals and multicomponent NPs, where batch variability and drug–botanical interactions can obscure efficacy and threaten safety in oncology polypharmacy [162,163,164,165]. Despite a robust development pipeline, regulatory approvals are rare: the FDA has received over 800 pre-IND and IND submissions for botanical products, yet only two botanical new drug applications (NDAs) have been approved—Veregen (sinecatechins; a standardized green tea extract topical) and Mytesi/Fulyzaq (crofelemer; a *Croton lechleri*-derived proanthocyanidin mixture for HIV-associated diarrhea) are the only FDA-approved botanical NDAs to date—illustrating the gap between mechanistic potential and regulation [166]. NP development faces hurdles, including scalable sourcing, compositional control, and ADMET/PK optimization, which hinder clinical progress [30].

### 9.1. Patient Stratification and Responder Enrichment

Metastasis and resistance mechanisms, including EMT plasticity, stem-like states, TGF-β signaling, DNA damage response rewiring, and immune exclusion, vary among patients and within tumors. Therefore, AI results should be presented not only as “targets” but also as pathway states to improve patient stratification. A simple workflow involves: (1) proposing a pathway or state hypothesis—such as TGF-β/SMAD-high EMT, Wnt/β-catenin stem-like program, or CXCR4-high stress-adapted state; (2) linking it to a measurable biomarker panel, such as immunohistochemistry/proteomics, bulk RNA signature, or circulating markers; and (3) applying this rule prospectively to select likely responders for trials. Trial designs should align with the biomarker’s validation stage, progressing from analytical to clinical validity and ultimately clinical utility, to prevent overestimating its predictive power based only on mechanistic or retrospective evidence.

### 9.2. Biomarker-Guided NP Selection and On-Treatment Monitoring

To validate the “AI → mechanism → outcome” framework in clinical settings, each NP should be supported by: (i) a predictive biomarker that indicates who should receive treatment, and (ii) a PD biomarker that demonstrates whether the targeted pathway is affected at tolerated doses. A practical template includes baseline predictive markers such as target abundance or pathway activity scores—like EMT, stemness, or DDR modules—along with a high target fraction, such as the CD133-high state or a CXCR4-high program. Early on-treatment PD markers involve pathway changes consistent with the mechanism, such as decreased phospho-nodes, altered transcription factor localization, or suppression of transcriptomic modules, measured through tumor biopsies when possible and supported by minimally invasive surrogates. Cellular techniques, like CETSA and TPP, enhance mechanistic insights by confirming protein engagement in cells beyond mere binding [147,167]. For early response assessment, serial circulating tumor DNA (ctDNA) dynamics can serve as exploratory PD/response endpoints in early NP trials; for example, early fluctuations in ctDNA have been linked to chemotherapy response and outcomes in metastatic colorectal cancer [168].

### 9.3. Regulatory and Development Pathway Considerations

Translational planning varies depending on whether the product is a purified single chemical, a defined multicomponent mixture, or a botanical drug. Each approach has specific requirements for identity, batch consistency, impurity profiling, stability, and comparability across clinical phases [166]. A common challenge in NP translation is establishing mechanistic plausibility without sufficient CMC control, which is crucial for ensuring consistent active composition exposure across studies. Additionally, in oncology, polypharmacy heightens the risk of DDIs; early screening for CYP/transport modulation should be incorporated into the design process, potentially using AI triage—such as penalizing candidates predicted to strongly impact CYP3A4 or ABC transporters [162,163,164,165].

### 9.4. Formulation, Pharmacokinetics, and Exposure Control

Many NPs fail in clinical settings due to issues such as poor solubility, unpredictable or low bioavailability, rapid clearance, or exposure levels that do not reach pharmacologically effective concentrations. To improve success rates, NP development should start with a well-defined exposure strategy that links the intended mechanism to achievable systemic and tumor concentrations. This includes clarifying whether efficacy relies on concentration, the duration above a certain threshold, or metabolites. Formulation approaches encompass lipid-based systems (such as SEDDS), prodrugs or salt forms of purified compounds, and nanoformulations when appropriate, to enhance stability and delivery [169,170].

### 9.5. Scalability, Supply Chain, and Quality Systems

Scalability is a key consideration for NP-derived therapeutics, as it affects their practical use. Major challenges include variability in source material due to geography, season, and cultivation, as well as the risk of adulteration. Additionally, validated analytical fingerprints and specifications for active compounds and impurities are required under GMP standards [30,166]. Experiences with high-value NPs, such as paclitaxel, show that sourcing and manufacturing scalability often delay clinical development until semi-synthetic or biotechnological approaches are adopted [171]. When supply is limited, AI should focus on candidates that can be produced through achievable synthetic, semi-synthetic, or cultivable/fermentable methods [30].

### 9.6. Practical Integration into Trial Design

To make AI-guided mechanistic hypotheses more understandable as clinical signals, early trials should include: (i) biomarker-based enrollment, (ii) predefined early PD confirmation windows, (iii) rational drug combinations targeting resistance, and (iv) clear failure criteria, such as not reaching exposure levels or not observing a PD shift within a specific window, to prevent ambiguous results. Using master-protocol designs—like basket, umbrella, or platform trials—can efficiently evaluate pathway-specific agents across biomarker-defined groups while ensuring statistical and operational consistency [172,173,174].

## 10. Current Limitations

While the preceding sections have outlined advances in AI-driven NP discovery, several fundamental challenges must be acknowledged, and promising emerging methodologies merit discussion. Several persistent barriers continue to impede the translation of AI methodologies into impactful outcomes in NP discovery. Data standardization remains a critical limitation: NPs’ bioactivity data are fragmented across disparate literature sources and databases, with inconsistent compound identifiers, variable assay conditions, and heterogeneous activity metrics that complicate aggregation and model training. The absence of unified data standards hampers the construction of comprehensive training sets and limits model generalizability across different data sources [175,176,177].

Stereochemical annotation is a substantial bottleneck for AI/chemoinformatics in natural-product discovery. Because many aggregated NP and bioactivity resources include entries with undefined/unknown stereocenters and ambiguous E/Z configurations, stereochemical variants may be collapsed or under-specified, limiting the reliability of stereochemistry-sensitive modeling. Curated resources like COCONUT still show a significant portion of molecules with stereocenters but missing absolute configuration, reflecting upstream source limitations. For example, COCONUT 2.0 reports 73,563 out of 695,133 (about 10.6%) unique structures with stereocenters but undefined absolute configuration in its September 2024 release [85]. When stereochemistry is uncertain, ML training labels may conflate stereoisomers with potentially different bioactivities [178,179]. In docking/virtual screening, stereoisomeric state can materially affect ranking and pose fidelity, motivating explicit stereoisomer enumeration when stereochemistry is uncertain [180]. Best practices involve: (i) flagging entries with undefined stereocenters during preprocessing, (ii) training models that are aware of stereochemistry and encode 3D configurations, and (iii) experimentally confirming the stereochemistry of hit compounds before optimization. Activity cliffs, defined as molecules that are structurally similar but have significantly different activities, present particular challenges for NP discovery because minor stereochemical differences can affect bioactivity. MaskMol is a knowledge-guided, self-supervised learning framework that generates detailed molecular representations to distinguish between activity cliff pairs [181]. This approach has successfully identified potential EP4 inhibitors for tumor therapy and has shown high accuracy across 20 macromolecular targets. In NP screening, where stereoisomeric and regioisomeric variants often demonstrate varied activities related to metastasis and resistance mechanisms, activity cliff-aware models can improve the prioritization of promising candidates.

Dereplication, which involves the early identification of known compounds in discovery workflows, is a significant challenge in AI-driven NP screening that is often overlooked. Without effective dereplication, compounds identified computationally may already be known, resulting in wasted validation effort. Key strategies for dereplication include: (i) cross-referencing databases such as COCONUT, LOTUS, and commercial NP libraries; (ii) matching spectroscopic data (MS/MS, NMR) against reference spectra; and (iii) utilizing computational tools like SIRIUS + CSI:FingerID (SIRIUS v6.3.3) and for structure verification [182,183]. AI can enhance the dereplication process by learning embeddings that reveal structural similarities beyond traditional fingerprint methods.

Combining metabolomics with AI-powered NP discovery offers strong capabilities for the early detection of known compounds and for verifying the source organism. Tandem mass spectrometry-based molecular networking enables rapid comparison of NP profiles with reference data, thereby distinguishing new compounds from known ones before extensive validation [184]. This approach has successfully identified cancer-related NP classes, including COX-2-inhibitory saponins from plants, using LC-MS/MS and molecular docking [185]. For AI-driven NP screening targeting metastasis and chemoresistance, integrating molecular networking as a post-analysis filter helps avoid rediscovering known active compounds. It supports annotating structural analogs with improved features. Additionally, the combination of comparative genomics, metabolomics, and molecular networking has proven effective in discovering new bioactive NPs from microorganisms, such as anticancer lipopeptides from *Nocardia* species [184]. These integrated methods demonstrate how AI, when combined with complementary analytical techniques, can accelerate NP discovery while preserving novelty.

Additionally, a significant challenge when using AI models trained mainly on synthetic drug-like compounds for NP screening is domain shift—the drop in performance when query molecules differ significantly from the training data. Yang et al. demonstrated through extensive testing on 35 datasets that, while graph CNNs generally outperform fixed molecular descriptors, their ability to generalize varies with new chemical scaffolds outside the training set [186]. This is especially important for NP discovery, as NPs occupy unique regions of chemical space characterized by higher sp^3^ content, greater stereochemical complexity, and scaffold structures rarely observed in synthetic datasets. In applications related to metastasis and chemoresistance, where NP scaffolds can offer distinct polypharmacological profiles not achievable with typical drug-like molecules, researchers should carefully assess a model’s applicability domain before trusting its predictions. Scaffold-based data splitting, rather than random splitting, provides more accurate estimates of future performance in NP screening efforts.

A key, but often overlooked, aspect of computational NP discovery is synthetic accessibility—the practical ability to produce sufficient quantities of hits for biological testing and further development. While NPs can be extracted from natural sources, limited supplies can obstruct drug development. To solve this, AI-driven retrosynthetic planning tools have been created. For instance, BioNavi-NP, a deep learning system that predicts NP biosynthetic pathways, employs end-to-end transformer neural networks trained on organic and biosynthetic reactions [187]. It achieved a 90.2% success rate in finding pathways for test NPs and recovered biosynthetic building blocks for 72.8% of compounds, roughly 1.7 times more accurate than traditional rule-based approaches. For NPs that serve as metastasis or chemoresistance modulators, these tools can help determine whether to pursue total synthesis, semi-synthesis from biosynthetic intermediates, or fermentation. Furthermore, general retrosynthesis tools such as ASKCOS can analyze synthetic routes for NP-inspired analogs, supporting optimization when the supply of natural sources is limited [137]. Including synthetic accessibility scores as a key filter in AI prioritization workflows is crucial to ensure that computational and experimental efforts are focused solely on candidates that are practically feasible. This evaluation should be performed early in the screening process—preferably right after or alongside ADMET filtering—rather than after identifying hits. For compounds with limited natural sources and no established biosynthetic methods, forward chemical retrosynthetic analysis with tools like ASKCOS [137] or SAScore-based filtering [136] should be used to determine if the compound or similar analogs can be produced at a scale suitable for preclinical development.

### 10.1. Data Quality, Bias, and Imbalance in AI-Based Natural-Product Discovery

Data quality and systemic bias significantly influence the dependability and reproducibility of AI-driven NP discovery. Unlike standardized prospective screening efforts, evidence of NP bioactivity mostly comes from retrospective data collected across various assay formats, endpoints, and reporting methods, causing notable variability in labels and covariates. Standards such as MIABE define critical metadata—entity identity, purity, assay context, endpoint definitions, and outcomes—but, because their use is voluntary and inconsistent across studies, they pose challenges for data harmonization and interpretation [175].

#### 10.1.1. Dataset Imbalance, Scarcity of Negatives, and Selection Bias

A pervasive limitation in public bioactivity databases, including NP-enriched subsets, is the imbalance between classes and the presence of non-random missing data—specifically, which compounds are tested and whether results are published or curated. Positive results are more frequently reported and integrated, which can bias decision boundaries and lead to inflated performance metrics when using non-stratified splits. Studies have shown systematic differences between models trained on public data and on proprietary industry data, with models trained on public data often overpredicting actives on out-of-distribution data [188]. This problem is compounded by the lack of confirmed inactive controls (true negatives) for many targets and assays, underscoring the importance of careful problem framing—such as treating “untested” compounds as unlabeled rather than negative—and adopting strategies like cost-sensitive learning, calibrated probability estimates, and uncertainty quantification.

#### 10.1.2. Incomplete and Heterogeneous Bioactivity Annotation

Public NP bioactivity entries often omit key metadata, including assay protocols, cell context, concentration ranges, counter screens, stereochemical details, mixture composition, and consistent activity units or thresholds. Curated resources help lessen—though not eliminate—this variability by standardizing the relationships between targets, assays, and measurements, normalizing units when feasible, and flagging ambiguous records [176].

#### 10.1.3. Scope Limitations of Drug-Centric Resources and the Need for NP-Specific Integration

Curation-focused platforms such as ChEMBL improve consistency by explicitly linking assays and targets and offering structured activity data. However, their primary focus remains on drug discovery, with an emphasis on bioactive small molecules with drug-like properties. Recent updates have added features relevant to NPs, including NP flags and NP-likeness scores. Despite these improvements, they do not fully capture the diversity of NP chemical space, which includes high stereochemical complexity, glycosylation patterns, macrocycles, and organismal origins [189]. Therefore, effective NP modeling often relies on specialized resources such as COCONUT, LOTUS, and the NPs Atlas, which broaden structural coverage and provide provenance information [25,86,190].

#### 10.1.4. Redundancy Across NP Databases and Leakage-Aware Evaluation

NP resources frequently combine data from overlapping primary sources, which can introduce risks such as redundancy—where structures are duplicated or nearly identical—and data leakage if similar compounds appear in both the training and test sets. This leakage can significantly inflate the model’s perceived performance and obscure important failure modes for future NP discovery. Best practices include (i) thorough deduplication, using normalized InChIKey and standardizing salts and tautomers before data splitting, and (ii) evaluating model performance with out-of-distribution (OOD) split methods, such as scaffold- or cluster-based partitions, instead of random splits. Scaffold splitting is particularly common in molecular ML benchmarking because it provides a more realistic challenge for generalization [191]. To better control leakage, dedicated splitters that implement similarity-aware partitioning can reduce train–test overlap and yield more accurate performance estimates [192].

#### 10.1.5. Reporting and Methodological Recommendations

To directly mitigate reproducibility risks in AI-based NP discovery—especially for metastasis and chemoresistance areas—the following practices should be documented and/or adopted as standard:

1. Provenance and preprocessing of data, including structure standardization, stereochemistry management, handling mixtures, unit normalization, and thresholding rules.
2. Harmonization of assay endpoints based on minimum-information standards (MIABE), where feasible [175].
3. Leakage-aware data splitting techniques such as deduplication, scaffold, cluster, or OOD splits, combined with explicit similarity constraints [191,192], and external validation.
4. Addressing data imbalance through methods like class weighting, decision threshold calibration, uncertainty estimation, and explicit handling of unlabeled negatives. Report both precision-recall metrics and AUROC.
5. Evaluation of bias and data shift, including performance gaps between public and proprietary datasets, and validation across different times or sources to prevent overly optimistic claims [188].

Together, these strategies help prevent performance estimate inflation, clarify the domains of applicability, and enhance the credibility of AI-identified NP candidates for further mechanistic and translational research.

### 10.2. Translational Bottlenecks: From Computational Prediction to Preclinical Validation

The limited number of Level A (in vivo-validated) AI-driven NP studies highlights ongoing translational challenges despite advances in computational prioritization. Progress from Level C/B to Level A often stalls at four key points: (i) compound materialization and quality control, (ii) target engagement and causal mechanisms, (iii) pharmacokinetics and exposure at the disease site, and (iv) model selection that accounts for metastasis and chemoresistance biology. Many computationally prioritized NPs remain “database entities” until supply, dereplication, and structural confirmation are complete; issues such as batch variability, isomerism, impurities, and reporting inconsistencies can reduce apparent activity during replication. In addition, AI and network-based methods frequently identify pathways or modules without direct evidence of target engagement in cells or tissues, which is crucial for establishing mechanisms. Cellular target engagement assays and label-free techniques exemplify the essential evidence that is often missing in Level B studies [147,193]. Moreover, NPs usually face developability issues—such as poor solubility, permeability, metabolic stability, or high plasma protein binding—so in vitro potency does not always ensure adequate tumor exposure. Therefore, permeability screening is commonly employed early in small-molecule development [193]. Furthermore, hypotheses about metastasis and resistance generally require models beyond standard subcutaneous xenografts. Orthotopic and metastatic models better mimic organ-specific microenvironments and dissemination but are more complex, less scalable, and necessitate rigorous study design [194].

A practical approach is to postpone resource-intensive in vivo studies until candidate compounds are de-risked mechanistically and pharmacologically. After computational consolidation (Level C)—which involves methods like network pharmacology, CPI prediction, and perturbational signatures when available, along with leakage-resistant evaluation and uncertainty/applicability analysis—compound QC (from C to early B) should confirm identity and batch consistency using techniques like LC–MS/NMR and validate purity for the intended use. Target engagement (early B) should then verify that the selected targets are active in a biological context, using complementary biophysical and cellular methods (e.g., CETSA, SPR/BLI/ITC, chemoproteomics when feasible). CETSA supports target engagement in cells and tissues [147] while DARTS offers a complementary label-free approach [195].

**Figure 5:**
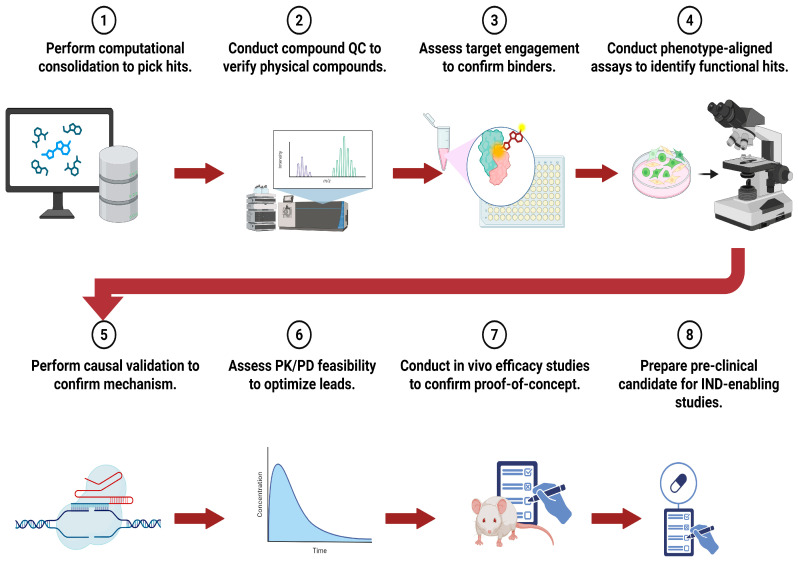
Stage-gated workflow for developing AI-selected NPs from initial computational ideas to preclinical validation. The scheme outlines an eight-step, de-risking process aimed at increasing the likelihood of obtaining strong Level A evidence by systematically screening candidates and deepening mechanistic insights. (1) Computational methods combine diverse in silico signals—such as CPI prediction, network pharmacology, perturbation signatures, ADMET risks, and uncertainty checks—to identify high-confidence hits. (2) Compound production and quality control (QC) verify physical availability and identity through techniques like dereplication and structure/purity analysis using LC–MS and NMR; batch consistency is checked for isolates, extracts, or formulas. (3) Target engagement assesses the plausibility of binding and interacting within cells, using assays like SPR/BLI/ITC, CETSA/DARTS, and chemoproteomics where relevant. (4) Functional assays, aligned with phenotypic outcomes, evaluate metastasis and chemoresistance factors beyond cell viability, including invasion, migration, EMT/partial-EMT, CSC-like cells, drug efflux, and DNA damage/repair markers—using 2D and 3D models such as spheroids, organoids, or co-cultures. (5) Causal validation determines if the target or pathway is essential and sufficient through perturbation and rescue techniques like CRISPR KO/KD/CRISPRi, drugs, epistasis, or rescue experiments. (6) PK/PD studies confirm whether adequate exposure levels are achievable and link exposure to pathway changes, utilizing PK and PD biomarkers, and optimizing formulations if necessary. (7) In vivo efficacy tests provide proof-of-concept in relevant models—such as syngeneic or immune-competent models for TME hypotheses, orthotopic or metastasis models for spreading, PDX or PDOX models for patient relevance, and combination therapies for resistance issues. (8) Preclinical candidate selection integrates efficacy, safety, and developability data to choose the best leads for IND-enabling studies. Created with BioRender.com.

Phenotypic confirmation (Level B) should focus on resistance- and metastasis-related endpoints beyond viability, such as invasion/migration, EMT/partial-EMT programs, CSC-like fractions, drug efflux activity, DNA damage/repair markers, and immune/TME readouts in co-culture if appropriate. Establishing mechanistic causality (from B to A) involves perturbation logic—using KO/KD/CRISPRi, pharmacologic antagonism, rescue, or epistasis—to demonstrate that the phenotype depends on the targeted pathway. PK/PD feasibility (Level A prerequisite) should show realistic exposure (stability, permeability, plasma binding) and connect exposure to pathway modulation via PD biomarkers, guiding formulation when solubility limits exposure. In vivo validation (Level A) requires selecting models that match the claim: immune-competent models for TME hypotheses, orthotopic/metastatic models for dissemination, and PDX/PDOX models where patient relevance is required. Notably, PDX establishment is time-consuming, and engraftment success varies; establishment can take months, and failure should not be assumed prematurely [196,197]. This explains why few candidates reach this stage. Finally, based on the previous limitation, we propose a stage-gated validation workflow (computational consolidation → compound QC → target engagement → phenotype-aligned assays → causal validation → PK/PD feasibility → in vivo efficacy) to de-risk candidates before committing to resource-intensive animal studies (Figure 5).

## 11. Future Directions

Several emerging developments could reduce current bottlenecks and greatly improve AI for NP discovery. To better guide the field, we categorize these advances and recommendations by their implementation horizon, separating near-term actionable priorities from medium- and long-term technological frontiers.

### 11.1. Near-Term Priorities: Validation, Benchmarking, and Translational Readiness

Given the current landscape, we recommend several priorities to accelerate AI-driven NP discovery for metastasis and chemoresistance. First, routine prospective validation should replace retrospective benchmarks, involving pre-registered, comprehensive studies in which AI-generated hypotheses are tested through orthogonal assays and failure modes are documented to minimize selection and reporting biases. Second, specific benchmarks for NPs are essential to assess how well models generalize across chemical space, focusing on features such as stereochemistry, increased sp^3^ content, and scaffold complexity, using standardized splits (e.g., scaffold or temporal splits) and community leaderboards for fair comparison. Third, standardized reporting and proper data management are critical, requiring minimum-information checklists for bioactive entities and FAIR-compliant metadata—covering identifiers, stereochemistry, assay protocols, endpoints, and provenance—to improve dataset interoperability, reproducibility, and model reliability. Lastly, fostering early translational collaborations involving medicinal chemistry, DMPK/ADMET triage, and developability criteria can reduce the risk of computational hits and increase the likelihood of advancing validated NPs beyond academic proof-of-concept [175,191,198,199]. Equally important is adopting explainable AI (XAI) as an early design requirement, rather than adding it later. Black-box models that rank NP candidates without clarifying their structural or mechanistic basis offer limited guidance for medicinal chemistry and may hinder regulatory approval. Explainable techniques—such as attention-based attribution, SHAP feature importance, and substructure reasoning with GNNs—highlight the molecular fragments and pharmacophoric features that influence predictions. For example, Wong et al. applied explainable graph algorithms to ensembles trained on over 39,000 compounds, identifying substructure rationales that led to the discovery of a novel antibiotic class active against MRSA [200]. A subsequent protocol provides a step-by-step, coding-free method to implement this explainable approach, making it more accessible to the NP discovery community [201]. Similar XAI workflows could be used in NP-based anti-metastasis research to identify which NP substructures drive activity against resistance targets such as EMT regulators, DDR kinases, or efflux transporters—supporting rational analog design and providing mechanistically interpretable evidence for biomarker-guided clinical application.

### 11.2. Medium-Term Advances: Foundation Models and Explainable Generative Design

Large-scale self-supervised “foundation” pretraining on molecular corpora can yield transferable representations that improve downstream performance under limited labels—an especially relevant property for NP settings where high-quality bioactivity data are sparse and heterogeneous [202,203]. In parallel, generative molecular design—including diffusion-based models and reinforcement learning (RL)—supports multi-objective optimization under explicit constraints (e.g., drug-likeness and synthesizability proxies, and sometimes target-conditioned objectives), enabling the proposal of NP-inspired analogs for subsequent triage and validation [204,205,206]. DiffMC-Gen demonstrates this strategy by integrating discrete and continuous denoising diffusion techniques with geometric neural networks to enhance binding affinity, drug-likeness, synthesizability, and toxicity simultaneously [207]. When used on cancer-related targets like LRRK2 and the GLP-1 receptor, it effectively generated new molecules with predicted biological activity and good drug-like properties. For NP-inspired designs targeting metastasis drivers, such multi-condition generation can help improve NP analogs while maintaining favorable ADMET profiles. However, these molecules still require experimental testing to confirm the predicted traits. Claims of improved binding affinity or ADMET should be interpreted primarily as optimization of predictive surrogates, requiring orthogonal validation. Moreover, preservation of “NP-like” character is best enforced via explicit metrics (e.g., NP-likeness scores or fragment constraints) rather than assumed NP-metric [87,208]. Integrating explainability into generative frameworks is a key short- to medium-term goal. Currently, these models primarily optimize predictive surrogates but rarely reveal which features or substructural motifs influence the generation process. Combining generative design with interpretability tools—such as attention visualization of molecular graphs or counterfactual explanations showing the minimal structural changes needed to alter a prediction—would help researchers verify whether generated analogs preserve pharmacologically relevant NP scaffolds or have moved into less accessible chemical spaces. This transparency is especially important when the generated molecules are used as leads for NP-inspired drug candidates targeting complex metastasis and resistance mechanisms, where understanding the underlying processes is critical throughout clinical development.

### 11.3. Long-Term Vision: Multimodal Data Integration

Finally, multimodal integration via knowledge graphs and transformer architectures can unify chemical structures, protein sequences, pathways, disease biology, and increasingly clinical-trial–linked evidence, improving target hypothesis generation and mechanism-of-action inference beyond single-modality models [209,210,211]. Achieving the full potential of multimodal AI in NP-based oncology requires integrating patient-level multi-omics data—such as transcriptomics, proteomics, and metabolomics—along with histopathological images and longitudinal clinical outcomes into a unified framework for compound prioritization. Analyzing deep learning in genomics and histopathology for precision oncology shows that, while single-modality approaches are well developed, integrating multiple data types remains a specialized area with significant untapped potential—especially for tasks such as predicting drug responses [212]. For AI-driven NP discovery, these multimodal models could simultaneously: (i) identify patient subgroups with molecular profiles indicating susceptibility to specific NP mechanisms (e.g., TGF-β/EMT-high tumors for NPs targeting epithelial plasticity), (ii) predict NP–target interactions and pathway effects based on compound structure, and (iii) estimate clinical response likelihood by linking predictions to outcome-labelled patient data. This would shift NP discovery from a solely compound-focused approach to a patient-centric, mechanism-informed pipeline, effectively connecting computational predictions with clinical translation. However, realizing this vision faces major data challenges: multi-omics datasets are dispersed and often incompatible across institutions; imaging features require domain-specific extraction before integration with molecular data; and clinical outcome data are limited, diverse, and confounded. Federated learning, which enables model training across distributed datasets without sharing sensitive patient information, offers a promising approach to achieving the scale and diversity needed for robust multimodal training. It has already demonstrated advantages in molecular discovery by improving model performance and broadening applicability through knowledge sharing among partners [213].

## 12. Conclusions

Cancer metastasis and chemotherapy resistance drive cancer mortality, causing most treatment failures and deaths. These multifactorial processes explain why single-target therapies often fail to control advanced cancers. NPs provide a valuable complement to therapy by suppressing EMT, reducing efflux transporters, inhibiting cancer stem cell renewal, and modulating immune responses—effects rarely seen with single agents. They are underrepresented in modern drug discovery due to structural complexity, data gaps, and pharmacokinetic challenges, hindering clinical development.

This review demonstrates that AI methodologies can address NP-specific challenges throughout the discovery process. GNNs and geometric deep learning grasp the stereochemical complexity vital for precise property prediction. Hybrid virtual screening methods that combine ligand-based enrichment with structure-based refinement generate mechanistic hypotheses while remaining computationally manageable. Network pharmacology, boosted by uncertainty-aware deep learning, enables systems-level target deconvolution suitable for NP polypharmacology. Importantly, the case studies—spanning computational hypotheses to in vivo validation—demonstrate that AI-driven NP discovery is practical, having yielded experimentally confirmed leads targeting metastasis and resistance mechanisms.

Despite this progress, notable obstacles remain. Stereochemical annotation in NP databases is frequently incomplete, which hampers the accuracy of model training and virtual screening. The domain shift between synthetic training data and NP query compounds diminishes prediction reliability, especially for novel scaffolds. Binding kinetics, increasingly recognized as vital for in vivo efficacy, remain largely out of reach for current AI models due to limited data. Additionally, there is a significant gap between computational prioritization and clinical translation; none of the AI-discovered NPs mentioned have yet undergone clinical trials. Historical attrition rates for NP-based candidates also indicate that computational success does not necessarily translate into therapeutic viability.

We suggest three key priorities that can drive advances in AI-driven NP discovery: First, the development of NP-specific benchmarks to assess model performance on complex, scaffold-diverse compounds using temporal and scaffold-based splits that mimic real discovery scenarios. Second, prospective validation studies should replace retrospective benchmarks and openly report both successes and failures to demonstrate method effectiveness. Third, translational factors should be incorporated early in the process as standard practice rather than retroactively. Specifically, forward synthetic accessibility must be considered a primary go/no-go criterion during initial screening, alongside ADMET predictions. This approach helps identify promising computational NPs that are practically inaccessible, allowing them to be deprioritized or redirected toward analog design before significant resources are committed. These suggestions, if applied, can reduce the loss of promising but practically challenging candidates. The merging of advanced AI with NP pharmacology offers not a shortcut but a structured framework for managing the complexity of NP chemical space and multifactorial cancer biology. Achieving this will require ongoing collaboration among computational scientists, NP chemists, and translational researchers, ensuring AI hypotheses are thoroughly tested and validated discoveries progress toward clinical trials for patients with treatment-resistant cancers.

## Data Availability

No new data were created or analyzed in this study. Data sharing is not applicable to this article.

## Abbreviations

**Abbreviation**	**Full Form**
ABC	ATP-binding cassette
ABCB1	ATP-binding cassette subfamily B member 1 (P-glycoprotein)
ABCC1/MRP1	ATP-binding cassette subfamily C member 1/multidrug resistance protein 1
ABCG2/BCRP	ATP-binding cassette subfamily G member 2/breast cancer resistance protein
ADMET	Absorption, distribution, metabolism, excretion, and toxicity
AI	Artificial intelligence
ALDH	Aldehyde dehydrogenase
AUC	Area under the curve
AUROC	Area under the receiver operating characteristic curve
BEAR	Bioactivity-based deep learning virtual screening method
BERT	Bidirectional encoder representations from transformers
BLI	Biolayer interferometry
BPYCF	Bupi Yichang Formula
CD44	Cluster of differentiation 44
CD133	Cluster of differentiation 133 (also known as PROM1)
CETSA	Cellular thermal shift assay
CFR	Conformalized fusion regression
ChEBI	Chemical Entities of Biological Interest
ChEMBL	Chemical database of bioactive molecules (EMBL-EBI)
CMap	Connectivity Map
CMC	Chemistry, Manufacturing, and Controls
CNNs	Convolutional neural networks
COCONUT	COlleCtion of Open Natural prodUcTs
COX-2	Cyclooxygenase-2
CPI	Compound–protein interaction
CRC	Colorectal cancer
CRISPR	Clustered regularly interspaced short palindromic repeats
CRISPRi	CRISPR interference
CSCs	Cancer stem cells
CSatDTA	Convolutional self-attention drug–target affinity model
ctDNA	Circulating tumor DNA
CXCR4	C-X-C chemokine receptor type 4
CYP	Cytochrome P450
CYP3A4	Cytochrome P450 family 3 subfamily A member 4
DARTS	Drug affinity responsive target stability
DCFME	Deep Collaborative Filtering with Multi-Embeddings
DDI	Drug–drug interaction
DDR	DNA damage response
DEGs	Differentially expressed genes
DiffDock	Diffusion-based molecular docking model
DimeNet	Directional Message Passing Neural Network
DMPK	Drug metabolism and pharmacokinetics
DMTA	Design–Make–Test–Analyze
DNA	Deoxyribonucleic acid
DTI	Drug–target interaction
DUD-E	Database of Useful Decoys-Enhanced
DYRK1A	Dual-specificity tyrosine phosphorylation-regulated kinase 1A
E(3)	Euclidean group in three dimensions
ECM	Extracellular matrix
EDL	Evidential deep learning
EGFR	Epidermal growth factor receptor
eIF2A	Eukaryotic translation initiation factor 2A
ELISA	Enzyme-linked immunosorbent assay
EMT	Epithelial-to-mesenchymal transition
EP4	Prostaglandin E2 receptor 4
EviDTI	Evidential deep learning-based drug–target interaction model
FAIR	Findable, Accessible, Interoperable, and Reusable
FDA	Food and Drug Administration (U.S.)
GCNN	Graph convolutional neural network
GEMMs	Genetically engineered mouse models
GemNet	Geometric Message Passing Neural Network
GEO	Gene Expression Omnibus
GEO-BERT	Geometry-based bidirectional encoder representations from transformers
GLP-1	Glucagon-like peptide-1
GMP	Good Manufacturing Practice
GNN/GNNs	Graph neural network(s)
GNINA	Deep learning-based molecular docking tool
GROVER	Self-supervised message passing transformer
GSEA	Gene set enrichment analysis
GSDMD	Gasdermin D
HPLC–MS	High-performance liquid chromatography–mass spectrometry
HQHPD	Huangqin Houpo decoction
HSP90	Heat shock protein 90
HTI-286	Hemiasterlin analog (synthetic tubulin inhibitor)
IC_50_	Half-maximal inhibitory concentration
IND	Investigational new drug
InChIKey	IUPAC International Chemical Identifier hash key
ITC	Isothermal titration calorimetry
K^d^	Equilibrium dissociation constant
K_i_	Inhibition constant
KO/KD	Knockout/knockdown
k_off_	Dissociation rate constant
k_on_	Association rate constant
KRAS	Kirsten rat sarcoma viral oncogene homolog
LASSO	Least absolute shrinkage and selection operator
LBVS	Ligand-based virtual screening
LC–MS	Liquid chromatography–mass spectrometry
LC-MS/MS	Liquid chromatography–tandem mass spectrometry
LINCS	Library of Integrated Network-based Cellular Signatures
LIT-PCBA	Literature PubChem BioAssay
LOTUS	Natural Products Online (Linked Open Unified Taxonomic database)
LOXL2	Lysyl oxidase-like 2
LRRK2	Leucine-rich repeat kinase 2
MCC	Matthews correlation coefficient
MD	Molecular dynamics
MDR	Multidrug resistance
MIABE	Minimum information about a bioactive entity
ML	Machine learning
MM-PBSA	Molecular mechanics Poisson–Boltzmann surface area
MMP2/MMP12/MMP13	Matrix metalloproteinases 2, 12, and 13
MoA	Mechanism of action
MolMVC	Molecular Multi-View Contrastive learning
MS/MS	Tandem mass spectrometry
NDA	New drug application
NF-κB	Nuclear factor kappa-light-chain-enhancer of activated B cells
NMR	Nuclear magnetic resonance
NOS3	Nitric oxide synthase 3
NP/NPs	Natural product(s)
NSCLC	Non-small cell lung cancer
OMIM	Online Mendelian Inheritance in Man
OOD	Out-of-domain
PAINS	Pan-assay interference compounds
PBPK	Physiologically based pharmacokinetic (modeling)
PD	Pharmacodynamics
PDX	Patient-derived xenograft
PDOX	Patient-derived organoid xenograft
P-gp	P-glycoprotein (ABCB1)
PI3K	Phosphatidylinositol 3-kinase
PK	Pharmacokinetics
PK-PD	Pharmacokinetic-pharmacodynamic
PPI	Protein–protein interaction
PROM1	Prominin-1 (CD133)
RF	Random forest
RIPK3	Receptor-interacting serine/threonine-protein kinase 3
RL	Reinforcement learning
RMSD	Root-mean-square deviation
RNA	Ribonucleic acid
ROS	Reactive oxygen species
SAR	Structure–activity relationship
SAScore	Synthetic accessibility score
SBVS	Structure-based virtual screening
SchNet	Continuous-filter convolutional neural network
SE(3)	Special Euclidean group in three dimensions
SEDDS	Self-emulsifying drug delivery system
SHAP	SHapley Additive exPlanations
SKiM	Serial KinderMiner
SMILES	Simplified Molecular Input Line Entry System
SPR	Surface plasmon resonance
STRING	Search Tool for the Retrieval of Interacting Genes/Proteins
SVM	Support vector machine
T-ALL	T-cell acute lymphoblastic leukemia
TCM	Traditional Chinese medicine
TCGA	The Cancer Genome Atlas
TGF-β	Transforming growth factor beta
TGFBR1	Transforming growth factor beta receptor 1
TGFBR2	Transforming growth factor beta receptor 2
TME	Tumor microenvironment
TNBC	Triple-negative breast cancer
TNFRSF10B	Tumor necrosis factor receptor superfamily member 10B
TOP2β	DNA topoisomerase II beta
TPP	Thermal proteome profiling
Tregs	Regulatory T cells
UAMRL	Uncertainty-aware multimodal representation learning
VEGFR-2	Vascular endothelial growth factor receptor 2
XAI	Explainable artificial intelligence
XGBoost	Extreme Gradient Boosting
XLF	XRCC4-like factor (DNA repair protein)
ZBM	Zhebeimu (Fritillaria thunbergii Miq.)
